# Oligomannose *N*-Glycans 3D Architecture and Its Response to the FcγRIIIa Structural Landscape

**DOI:** 10.1021/acs.jpcb.1c00304

**Published:** 2021-03-04

**Authors:** Carl A Fogarty, Elisa Fadda

**Affiliations:** Department of Chemistry and Hamilton Institute, Maynooth University, Maynooth, Kildare, Ireland

## Abstract

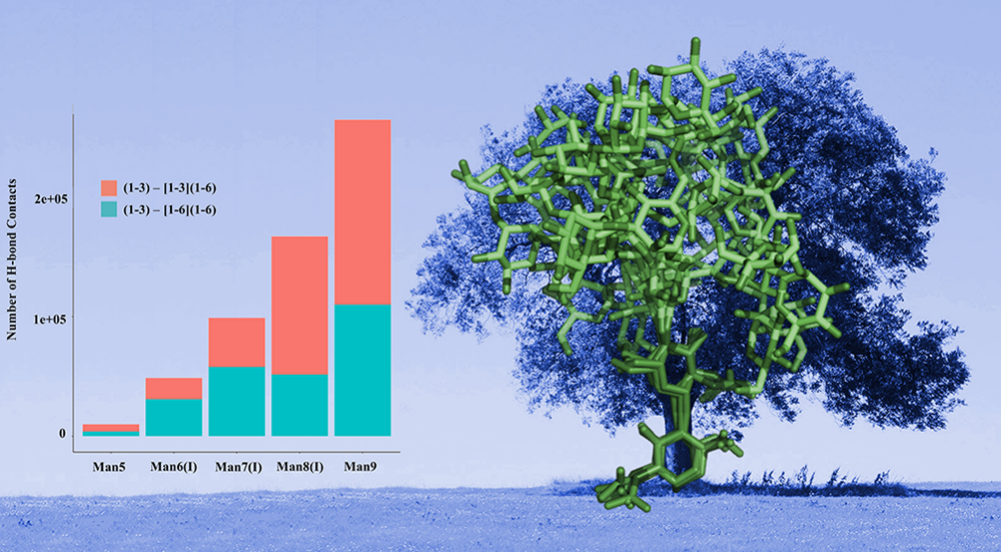

Oligomannoses are evolutionarily the oldest class of *N*-glycans, where the arms of the common pentasaccharide unit, i.e., Manα(1–6)-[Manα(1–3)]-Manβ(1–4)-GlcNAcβ(1–4)-GlcNAcβ1-Asn, are functionalized exclusively with branched arrangements of mannose (Man) monosaccharide units. In mammalian species oligomannose *N*-glycans can have up to 9 Man; meanwhile structures can grow to over 200 units in yeast mannan. The highly dynamic nature, branching complexity, and 3D structure of oligomannoses have been recently highlighted for their roles in immune escape and infectivity of enveloped viruses, such as HIV-1 and SARS-CoV2. The architectural features that allow these *N*-glycans to perform their functions are yet unclear, due to their intrinsically disordered nature that hinders their structural characterization. In this work we will discuss the results of over 54 μs of cumulative sampling by molecular dynamics (MD) simulations of differently processed, free (not protein-linked) oligomannose *N*-glycans common in vertebrates. We then discuss the effects of a protein surface on their structural equilibria based on over 4 μs cumulative MD sampling of the fully glycosylated CD16a Fc γ receptor (FcγRIIIa), where the type of glycosylation is known to modulate its binding affinity for IgG1s, regulating the antibody-dependent cellular cytotoxicity (ADCC). Our results show that the protein’s structural constraints shift the oligomannoses conformational ensemble to promote conformers that satisfy the steric requirements and hydrogen bonding networks demanded by the protein’s surface landscape. More importantly, we find that the protein does not actively distort the *N*-glycans into structures not populated in the unlinked forms in solution. Ultimately, the highly populated conformations of the Man5 linked glycans support experimental evidence of high levels of hybrid complex forms at N45 and show a specific presentation of the arms at N162, which may be involved in mediating binding affinity to the IgG1 Fc.

## Introduction

Complex carbohydrates (or glycans) are the most abundant biomolecules in nature. Within a human biology context, glycans coat cell membranes and protein surfaces, mediating a myriad of essential biological processes in health and disease states.^1−6^ N-glycosylation is one of the most abundant and diverse type of post-translational modification that can affect protein trafficking and structural stability and mediate interactions with different receptors.^6−11^*N*-glycan recognition and binding affinities are often highly specific to their sequence, intended as the types of monosaccharides, their stereochemistry, and branching patterns,^12^ a principle that has been successfully exploited in the development of glycan microarray technology.^13^

Molecular recognition is fundamentally dependent, among other considerations, on structural and electrostatic complementarity between the ligand and the receptor’s binding site. Within this framework, the prediction and characterization of glycan binding specificity are an extremely difficult task, due to their high degree of flexibility or intrinsic disorder, which hinders our ability to determine their 3D structure by means of experimental techniques. Indeed, glycans can only be structurally resolved in their entirety only when tightly bound to a receptor, thus when their conformational degrees of freedom are heavily restrained. Because of their inherent flexibility, free glycans can adopt different 3D structures within a weighted conformational ensemble, which cannot be determined with currently available experimental methods, although very promising steps forward have been recently made in advancing imaging techniques for single glycans.^14,15^

**Figure 1:**
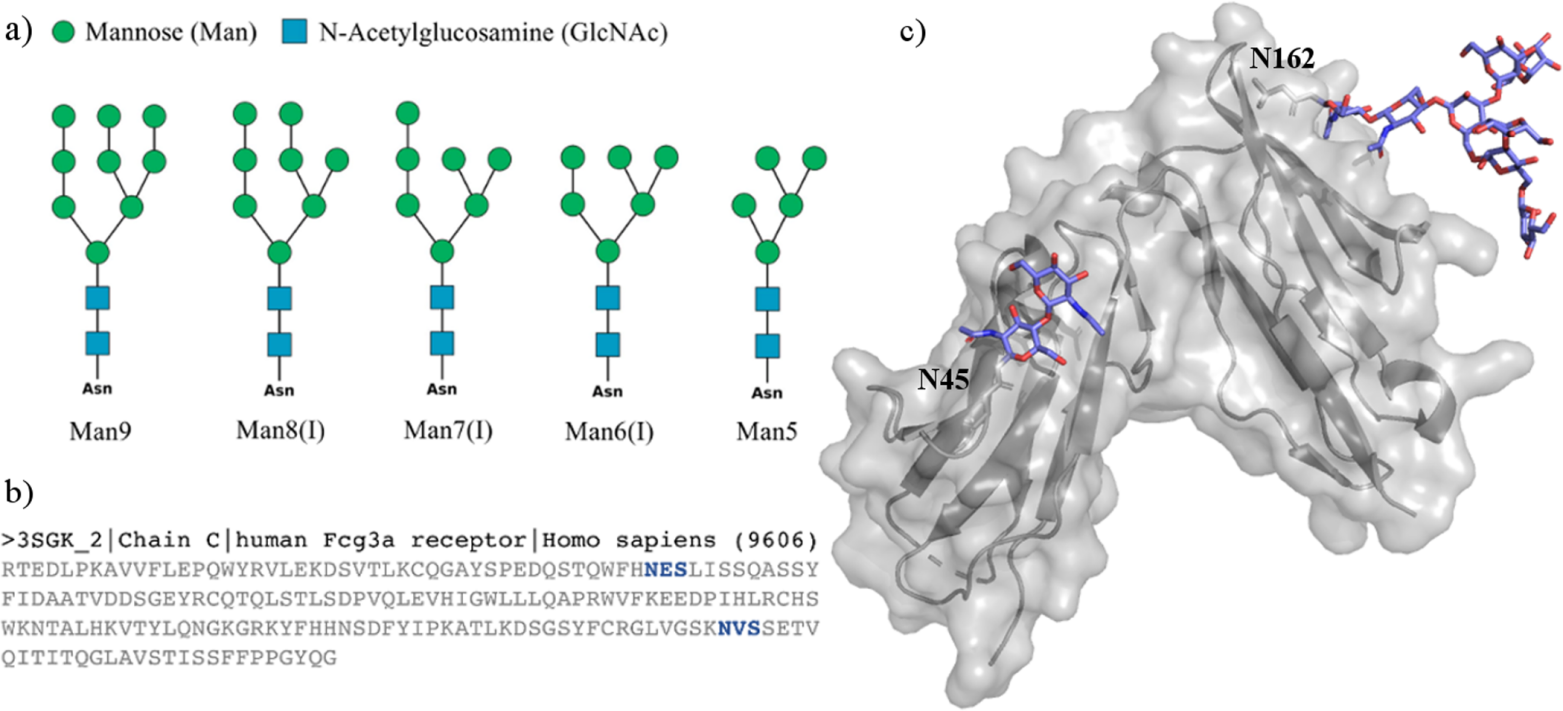
(a) SNFG representation^18^ of a subset of the oligomannose *N*-glycans discussed in this work. Man6/7/8(I) indicate specific positional isomers. For the complete list of isomers see Figure S.1. (b) Sequence of the human CD16a (FcγIIIa) of the PDB entry 3SGK(19) with the occupied sequons highlighted in blue. (c) Structure of the human CD16a (FcγIIIa) from PDB entry 3SGK with the resolved *N*-glycans at positions N45 and N162 represented with sticks. Image was rendered with VMD (http://www.ks.uiuc.edu/Research/vmd/). *N*-Glycan sketches were rendered with DrawGlycan (http://www.virtualglycome.org/DrawGlycan/).

High performance computing (HPC) molecular simulations can contribute a great deal toward our understanding of the relationships between glycans’ sequence, structure, and function. Indeed, conformational sampling through conventional and/or enhanced molecular dynamics (MD) schemes allows us to characterize the dynamic behavior of different glycoforms at the atomistic level of details. Within this context, for the past few years our lab contributed to the knowledge of *N*-glycans dynamics by providing information on their 3D architecture and relative flexibility from extensive MD-based conformational sampling.^16,17^ As an example, we have shown how the sequence (and branching) of complex *N*-glycans determines the 3D structure, which in turn drives their recognition.^16,17^ In this work we extend our data set of free (unlinked) *N*-glycans structures to the vertebrate oligomannose type, where, as shown in Figure 1, the common pentasaccharide unit, i.e., Manα(1–6)-[Manα(1–3)]-Manβ(1–4)-GlcNAcβ(1–4)-GlcNAcβ1-Asn, is functionalized by a branched arrangement of only Man units. In addition, we also determine how the protein surface landscape affects their conformational dynamics, which is a very important question in terms of its impact on molecular recognition and function while challenging to answer in absolute terms because of the site-specific character.

Oligomannoses are often defined as “immature” *N*-glycans, as they are processed toward complex functionalization in the Golgi^6^ and are not abundant in vertebrates. Nevertheless, these *N*-glycans are a common post-translational modification of viral envelope proteins expressed in human cell lines;^20,21^ for example, it is the prevalent type of glycosylation of the HIV-1 fusion trimer.^22−25^ Furthermore, an increase in large oligomannose-type N-glycosylation in humans has been linked to breast cancer progression^26−28^ and can occur where the protein landscape at the *N*-glycan site does not allow easy access to the required glycohydrolases and glycotransferases for further functionalization.^6,29,30^ Interestingly, recent work has shown that oligomannose *N*-glycans functionalizing CD16a low-affinity Fc γ receptors (FcγIIIa) determine an increase in IgG1-binding affinity by 51-fold,^31^ relative to the more common complex *N*-glycans,^32^ although the N-glycosylation composition varies depending on the glycosylation site.^32^

In this work we have studied the effect of the FcγIIIa protein surface landscape on the intrinsic conformational propensity of different oligomannose *N*-glycans we determined for the unlinked forms. Our results show that the two FcγIIIa N-glycosylation sites, N45 and N162, affect the oligomannose dynamics rather differently, in function of the structural constrains of the sites and of the 3D architecture of the glycan. More specifically, we find that the protein landscape affects the glycans conformational equilibrium by promoting structures that are complementary to it and not by actively changing their intrinsic architecture. Indeed, all the 3D conformers observed in the analysis of the bound oligomannoses are always identified in the simulations of the corresponding unlinked forms in solution, although in different populations. Interestingly, we also determined that the progressive elongation of the arms/branches promotes interarm contacts, where the Man9 3D architecture is almost entirely structured with interacting arms. Finally, these findings fit very well within the framework of our recently proposed “glycoblocks” glycans structure representation,^16^ whereby groups of specifically linked monosaccharides within *N*-glycans represent independent structural elements (or glycoblocks), whose exposure, or presentation in function of the particular protein landscape, drives molecular recognition.

## Computational Method

All 12 oligomannose starting structures, shown in Figures 1 and S.1, were obtained with the GLYCAM carbohydrate builder online tool (http://www.glycam.org). For each of these oligomannoses, we built nine structures characterized by different combinations of the two α(1–6) torsions values. Complete topology and parameter files were generated with the tLEaP tool from version 18 of the AMBER software package,^33^ with the GLYCAM06-j1 parameter set^34^ to represent the carbohydrates and the TIP3P model^35^ for water molecules. Because our simulations do not involve the calculation of hydration or of binding free energies^36,37^ and also because of consistency with our previous work,^16,17^ we consider the choice of GLYCAM06-j1/TIP3P parameter set as appropriate. All simulations were run in 200 mM NaCl salt concentration, with counterions represented by AMBER parameters^38^ in a cubic simulation box of 16 Å sides. Long range electrostatic were treated by particle mesh Ewald (PME) with cutoff set at 11 Å and a B-spline interpolation for mapping particles to and from the mesh of order of 4. van der Waals (vdW) interactions were cut off at 11 Å. The MD trajectories were generated by Langevin dynamics with collision frequency of 1.0 ps^–1^. Pressure was kept constant by isotropic pressure scaling with a pressure relaxation time of 2.0 ps. After an initial 500.000 cycles of steepest descent energy minimization, with all protein/glycans heavy atoms restrained by a harmonic potential with a force constant of 5 kcal mol^–1^ Å^–2^, the system was heated in two stages, i.e., from 0 to 100 K over 500 ps at constant volume and then from 100 to 300 K over 500 ps at constant pressure. After the heating phase, all restraints were removed and the system was allowed to equilibrate for 5 ns at 300 K and at 1 atm of pressure. Production and subsequent analysis were done on 500 ns trajectories run in parallel for each uncorrelated starting structure, i.e., each conformer generated with GLYCAM-Web. Analysis was done using the cpptraj tool and with VMD^39^ (https://www.ks.uiuc.edu/Research/vmd/). The dihedral distributions from the trajectories were obtained in terms of kernel density estimates (KDE), with a smoothing parameter of [1000,1000], with the ks package in R and rendered with heat maps with RStudio (www.rstudio.com) in conjunction with the DBSCAN clustering algorithm. The highest populated conformers resulting from the analysis of Man5 and Man9 were then grafted in positions N45 and N162 of the FcgRIIIa (PDB code 3SGK) by structural alignment to the resolved chitobiose at N45 and at N162, see Figure 1c, to obtain two systems, one with only Man5 and the other with only Man9 at both positions. As a note, the structure of the *N*-glycan at N165 from the PDB structure is quite distorted with uncommon ring conformations of some of the monosaccharides, probably resulting from the fitting to the electron density; therefore it was disregarded and only the chitobiose was used for structural alignment. These systems were run in duplicates from uncorrelated starting structures with the same simulation protocol used for the free glycans. Production runs were extended to 1 μs for each trajectory for a total of 4 μs of cumulative sampling time. All simulations were run on NVIDIA Tesla V100 16GB PCIe (Volta architecture) GPUs on resources from the Irish Centre for High-End Computing (ICHEC) (www.ichec.ie).

## Results

We used conventional MD simulations, run in parallel for 500 ns from nine uncorrelated starting points,^16,17^ to characterize the 3D structure and dynamics of human oligomannose *N*-glycans, when unlinked; see Figures 1 and S.1. The effects of the protein on their intrinsic dynamics were studied on two models with Man5 and Man9 linked to the human FcγIIIa protein on the two N-glycosylation sites, namely, N45 and N162; see Figure 1c. This section is organized as follows, first we present the results obtained for the unlinked oligomannoses, starting with Man5 that we used as a reference to describe sequence-to-structure changes in the larger forms. The subset of representative isomers shown in Figure 1 is presented here for simplicity, while the complete analysis of all positional isomers with heat maps and tables is included as Supporting Information. The section concludes with the results obtained for Man5 and Man9 when linked to the FcγRIIIa.

**Figure 2:**
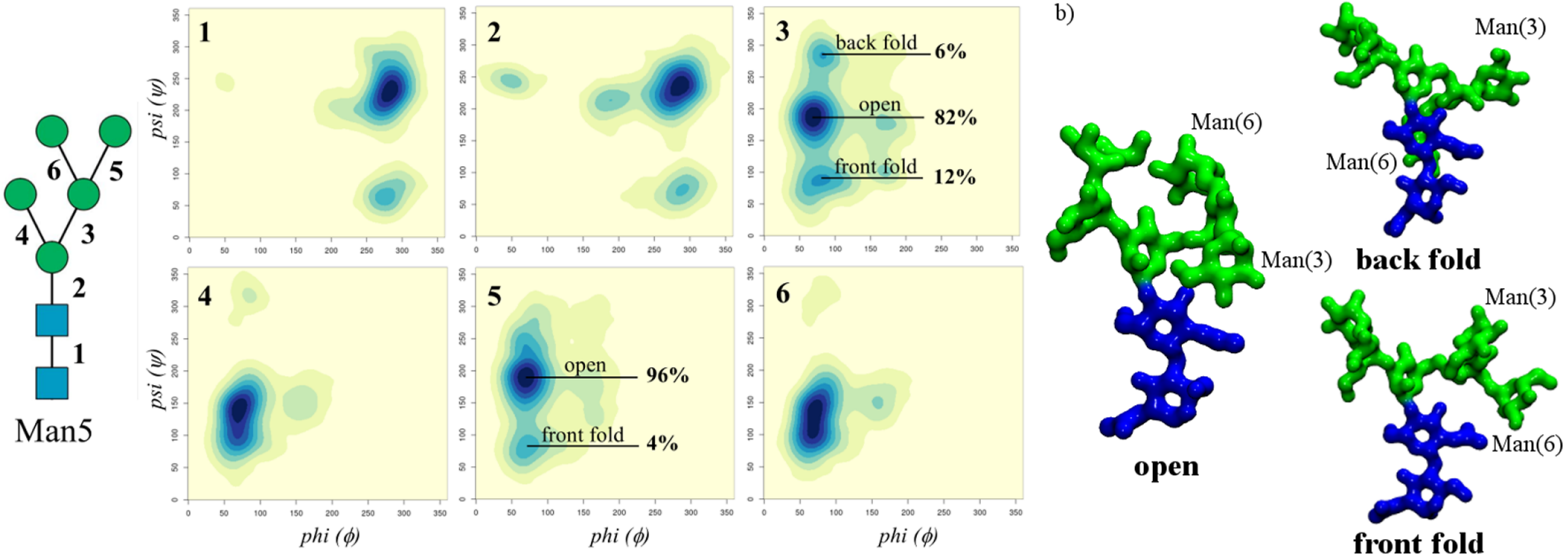
(a, Left) Man5 conformational analysis in terms of the ϕ and ψ torsion angles, with axes ranging from 0° to 360°. Each torsion is numbered as indicated on the left-hand side, and the heat maps are labeled in the top-left corner accordingly. (b) 3D structures of the dominant conformers determined by the flexibility of the (1–6) arm. The Man(3/6) labels indicate the position of the Man on the 3/6 branch on the (1–6) arm. Heat maps were made with RStudio (www.rstudio.com), and molecular models were rendered with VMD (http://www.ks.uiuc.edu/Research/vmd/). *N*-glycans are colored according to the SNFG convention.

**Figure 3:**
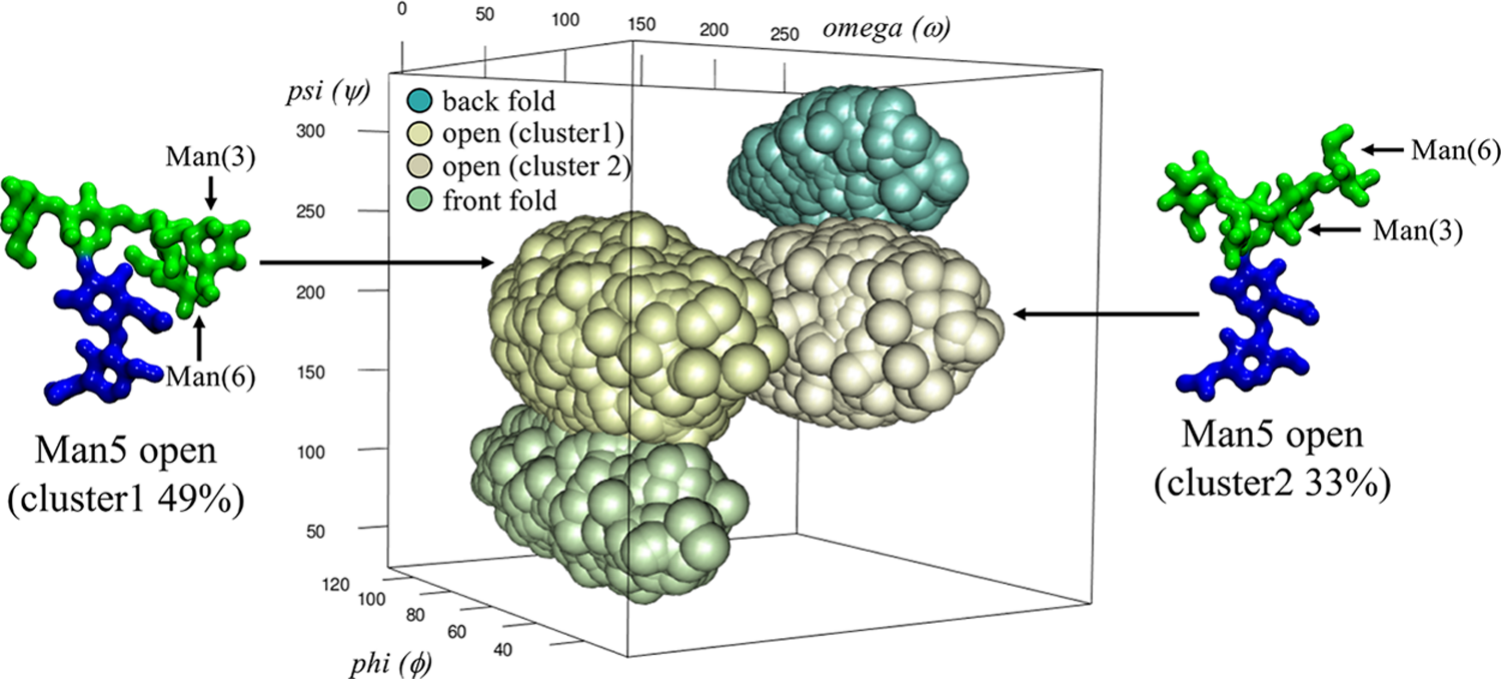
3D representation of the clustering analysis of the Man5 (1–6) arm torsion angles populations. While the back and front fold conformers have one set of values each for the ϕ, ψ, and ω torsions, the open conformation adopts two distinct orientations of the biantennary branch, namely, open (cluster 1) with a representative structure shown on the left-hand side and open (cluster 2) with a representative structure shown on the right-hand side. The relative positions of the Man(3)- and Man(6)-linked units are also indicated. The rgl package in RStudio (www.rstudio.com) was used to make the graphics. Molecular models were rendered with VMD (http://www.ks.uiuc.edu/Research/vmd/). *N*-glycans are colored according to the SNFG convention.

**Man5** is the simplest oligomannose found in vertebrates and the substrate of GlcNAc transferase I (GnTI), responsible for starting the *N*-glycan complex functionalization in the Golgi.^6^ As found for complex biantennary *N*-glycans,^16,17^ the Man5 chitobiose core and the following Manβ(1–4)-GlcNAc linkage are rigid with only one conformation significantly occupied, see Figure 2 and Table S.1, while the (1–3) arm adopts an outstretched conformation with flexibility in a range of 40° around the ψ torsion angle; see Figure 2 and Table 1. The Man5 (1–6) arm has a relatively more complex dynamics, hinging around the preferential “open” conformation,^16,17^ populated at 82%, where the Manα(1–3)-Man branch can be orientated toward the front of the page and the Manα(1–6)-Man branch toward the back of the page or *vice versa*. We also identified two alternative, less populated conformers, namely, a “front fold” (ϕ = 79°, ψ = 87°) with a relative population of 12% and a “back fold” (ϕ = 83°, ψ = −76°) with a relative population of 6%; see Figure 2 and Table 1. In the front fold the terminal Manα(1–6)-Man interacts through hydrogen bonds with the *N*-acetyl group of the second core GlcNAc, pushing Manα(1–3)-Man upward, while the back fold is stabilized by hydrogen bonds between the terminal Manα(1–6)-Man and both GlcNAc residues of the chitobiose; see Figure 2b. The open conformation can be further analyzed in terms of the α(1–6) linkage ω torsion angle that determines different orientations of the Manα(1–6)-Man and Manα(1–3)-Man branches relative to the core. As shown in Figure 3, there are two dominant conformers contributing to the open structure, open (cluster 1) populated at 48% with ω = 56° and open (cluster 2) populated at 33% with ω = −175°; see Table 1. The dynamics of the branches on the Man5 (1–6) arm follows the same pattern observed in the (1–3) and (1–6) arms, with only small differences dictated by their immediate environment. For example, the terminal Manα(1–6)-Man is found predominantly (80%) in the open (cluster 1) conformation, see Table 1, and does not show a back fold orientation.

**Table 1:** Torsion Angle Median Values of the Linkages in the Man5 (1–3/6) Arms^a^

Manα(1–6)-Man arm (3)	ϕ	ψ	ω	population (%)
open (cluster 1)	71 (11)	–172 (17)	56 (11)	49
open (cluster 2)	68 (10)	–175 (14)	–175 (13)	33
front fold	79 (16)	87 (13)	51 (10)	12
back fold	83 (9)	–76 (11)	–150 (10)	6

Manα(1–6)-Man branch (5)	ϕ	ψ	ω	population (%)
open (cluster 1)	70 (10)	–171 (16)	55 (10)	80
open (cluster 2)	70 (8)	–173 (13)	–81 (13)	6
open (cluster 3)	69 (8)	–120 (14)	–64 (10)	6
open (cluster 4)	70 (8)	–169 (17)	–164 (9)	4
front fold	71 (10)	83 (12)	48 (9)	4

Manα(1–3)-Man branch (6)	ϕ	ψ	population (%)
cluster 1	72 (9)	139 (5)	62
cluster 2	68 (10)	100 (10)	38

Manα(1–3)-Man arm (4)	ϕ	ψ	population (%)
cluster 1	72 (8)	142 (14)	74
cluster 2	69 (9)	100 (10)	26

a Standard deviation values are shown in parentheses, with relative populations obtained from clustering analysis. Angle values are in degrees. The number in parentheses in the first column indicates the linkages, as shown on the Man5 sketch in Figure 2.

**Figure 4:**
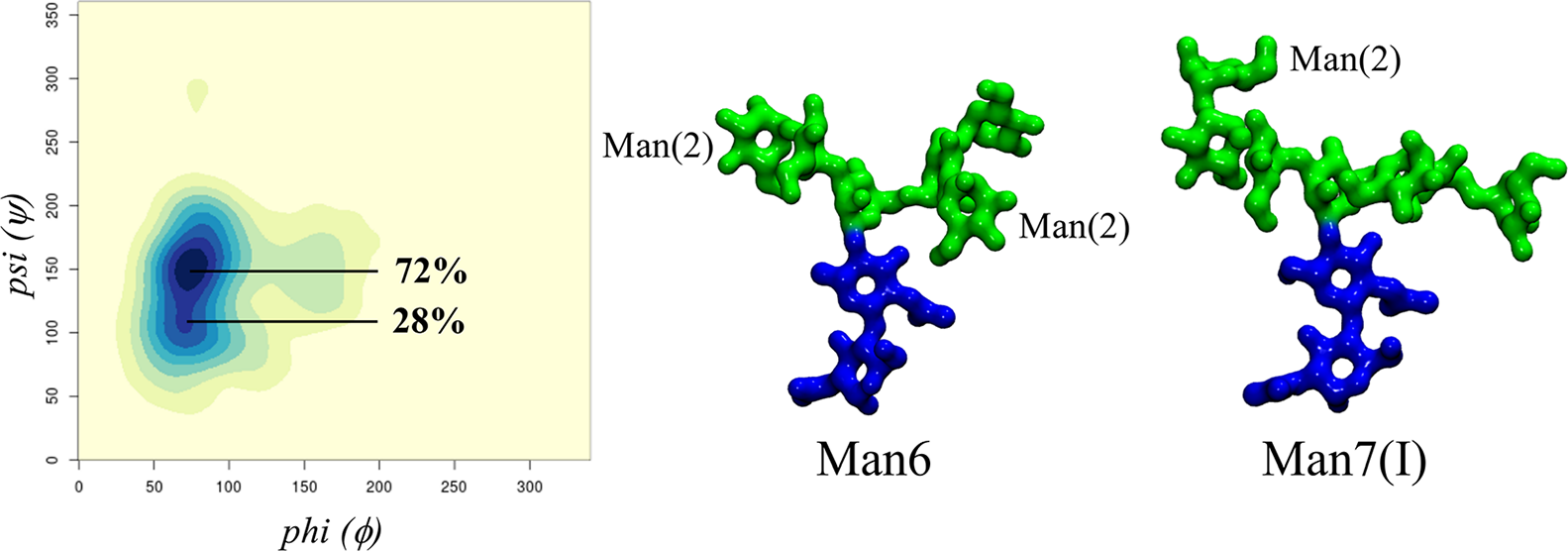
On the left-hand side, heat map representing the conformational analysis with corresponding populations of the first Manα(1–2)-Man linkage on the Man7(I) (1–3) arm, also representative of the corresponding linkage in the Man6 (1–3) arm. On the right-hand side, representative structures of Man6(I) and Man7(I) corresponding to the highest populated Manα(1–2)-Man linkage rotamers in the open (cluster 2) (ϕ = 71°, ψ = −172°, ω = −176°) conformation where the arms do not interact and the conformation of the Manα(1–2)-Man is more clearly visible. Heat maps were made with RStudio (www.rstudio.com), and molecular models rendered with VMD (http://www.ks.uiuc.edu/Research/vmd/). *N*-glycans are colored according to the SNFG convention.

**Man6(I) and Man7(I)** both have a longer (1–3) arm relative to Man5 with one and two Manα(1–2)-Man additional linkages, respectively. Note that different Man6/7 positional isomers exist, where the terminating Man can functionalize either branch on the (1–6) arm. We decided to highlight the Man6/7(I) positional isomers to present the effect of the elongation of the (1–3) arm in combination with a shorter (1–6) arm on the dynamics of the system and their role in enhancing contacts between the arms. A full set of all positional isomers is presented in the Supporting Information for completeness. As shown in Figure 4 and Tables S.2 and S.6, both Manα(1–2)-Man linkages occupy two conformers, one at (ϕ = 74°, ψ = 151°) and the other at (ϕ = 70°, ψ = 107°) with a relative population of 73% and 27% for Man 6, respectively, and one at (ϕ = 74°, ψ = 151°) and the other at (ϕ = 70°, ψ = 106°) with population of 76% and 24% for Man 7, respectively. As shown by the population analysis in Tables S.2 and S.7, the elongation of the (1–3) arm with Manα(1–2)-Man linkages does not affect the conformational propensity of the (1–6) arm relative to Man5, yet it slightly enhances the flexibility of the (1–3) arm, decreasing the population of the dominant conformer (ϕ = 72°, ψ = 142°) at 74% in Man5 down to 63% in Man7. Notably, the progressive elongation of the (1–3) arm with rigid Manα(1–2)-Man linkages determines an increase of the interarm contacts with both (1–6) branches relative to Man5, as discussed in the next subsection. These contacts are stabilized by a complex network of short-lived and interchanging hydrogen bonds that mostly involve the terminal residues of the arms. The open (cluster 1) conformation with α(1–6) torsion values (ϕ = 71°, ψ = −172°, ω = 56°) populated at 41% and 46% in Man6 and Man7, respectively, favors the formation of these arm–arm interactions; see Figure 6b. Notably, elongation of the (1–3) branch on the (1–6) arm in Man6 (II) and Man 7 (II) determines an increase of these arm–arm interactions that contributes to increasing the population of a previously negligeably populated “cluster 3” conformer, see Figures S.4 and S.7 and Tables S.3 and S.6.

**Figure 5:**
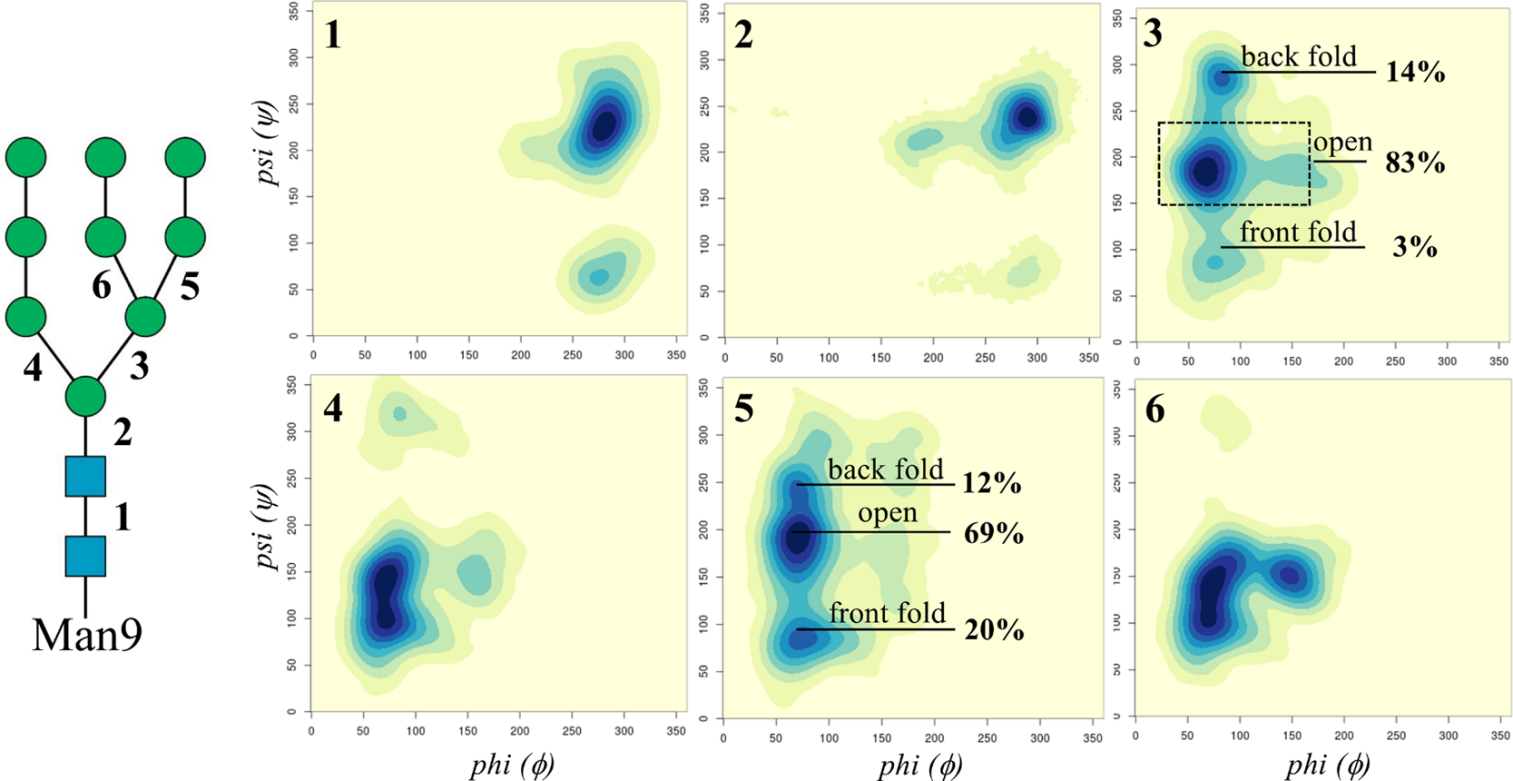
Man9 conformational analysis in terms of the ϕ and ψ torsion angles (ranging between 0° and 360°) explored during the 4.5 μs of cumulative MD sampling. Each torsion was numbered as indicated on the left-hand side sketch, and the heat maps have been labeled accordingly in the top left corner. Heat maps were made with RStudio (www.rstudio.com).

**Man8(I) and Man9** have further functionalizations of the (1–6) arm with one Manα(1–2)-Man linkage on the (1–3) branch for the Man8(I) positional isomer and an additional one on the (1–6) branch in Man9; see Figure 1. As seen for the other oligomannoses, in Man8(I) and Man9 the dominant conformation is with an open (clusters 1, 2, and 3) (1–6) arm, see Table 2 and Tables S.9 and S.13, with a slightly more pronounced preference for the back vs front fold in Man9, due to the interactions of the longer (1–6) branch with the chitobiose, see Figure 5 and Table 2.

**Table 2:** Torsion Angles Median Values of the Linkages in the Man9 (1–/6) Arms^a^

Manα(1–6)-Man arm (3)	ϕ	ψ	ω	population (%)
open (cluster 1)	79 (13)	–173 (16)	55 (12)	38
open (cluster 2)	66 (10)	–179 (13)	–177 (12)	37
open (cluster 3)	73 (9)	–157 (21)	–65 (11)	7
cluster 4	147 (9)	–172 (8)	47 (7)	1
front fold	78 (11)	87 (11)	46 (9)	3
back fold (cluster 1)	82 (8)	–74 (10)	–151 (10)	12
back fold (cluster 2)	76 (9)	–96 (10)	–75 (9)	2

Manα(1–6)-Man branch (5)	ϕ	ψ	ω	population (%)
open (cluster 1)	71 (10)	–169 (17)	55 (10)	57
open (cluster 2)	69 (10)	–179 (23)	–166 (11)	8
open (cluster 3)	71 (7)	–180 (15)	–73 (8)	4
front fold (cluster 1)	73 (12)	83 (12)	–164 (18)	10
front fold (cluster 2)	79 (16)	86 (12)	51 (10)	10
back fold	70 (7)	–118 (12)	–67 (10)	12

Manα(1–3)-Man branch (6)	ϕ	ψ	population (%)
cluster 1	76 (12)	139 (5)	55
cluster 2	70 (11)	100 (10)	26
cluster 3	148 (11)	151 (10)	18

Manα(1–3)-Man arm (4)	ϕ	ψ	population (%)
cluster 1	72 (9)	142 (15)	59
cluster 2	71 (11)	97 (11)	41

a Standard deviation values are shown in parentheses, with relative populations obtained from clustering analysis. Angle values are in degrees. The number in parentheses in the first column indicates the linkages, as shown on the Man9 sketch in Figure 5.

**Figure 6:**
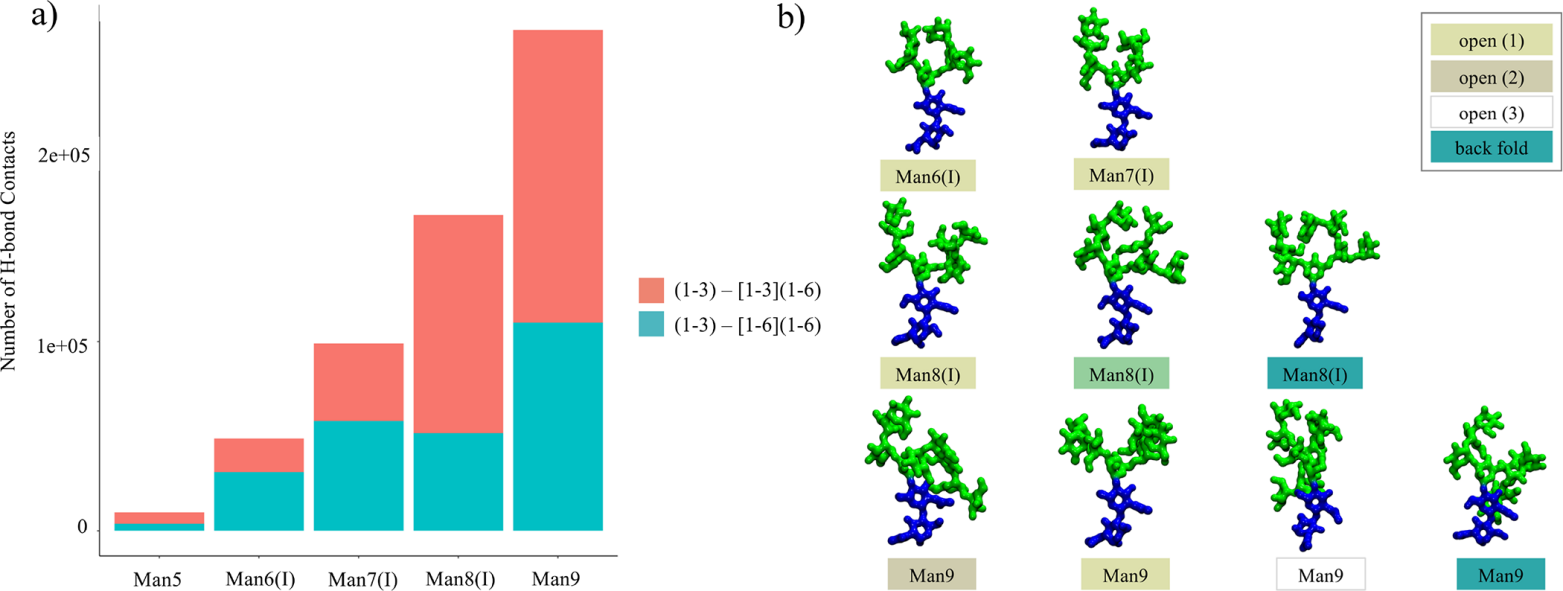
(a) Number of hydrogen bond contacts (distance threshold 4 Å between donor and acceptor atoms) counted over the 4.5 μs cumulative sampling for each oligomannose indicated on the *x*-axis. Contacts between the (1–3) arm and the (1–6) branch of the (1–6) arm are shown in cyan. (b) Representative snapshots from the MD simulations illustrating examples of the interarm contacts occurring within each conformational cluster. Different clusters are indicated by the colors in the legend on the top right-hand side, in agreement with the coloring scheme used in Figure 3. Histograms were made with RStudio (www.rstudio.com), and molecular models were rendered with VMD (http://www.ks.uiuc.edu/Research/vmd/). *N*-glycans are colored according to the SNFG convention.

The structures of all Manα(1–2)-Man linkages are the same as described for Man6(I) and Man7(I), yet the elongation of both branches on the (1–6) arm with relatively rigid Manα(1–2)-Man linkages determines structures with a high number of contacts between the two arms. Indeed, as shown in Figure 6, inter-arm contacts only occur within one conformational cluster in Man6(I) and Man7(I), namely, open (cluster 1); meanwhile in Man9, interactions between the arms are a feature of virtually all structural populations. These contacts are stabilized by complex networks of rapidly interchanging hydrogen bonds involving mainly the terminal monosaccharides on the arms and branches.

### FcγRIIIa-Linked Man5/9

The FcγRIIIa (CD16a) is a cell-bound receptor responsible for modulating antibody-dependent cellular cytotoxicity (ADCC) through its interaction with the IgG1 Fc region.^4^ Recent studies have shown that the FcγRIIIa glycosylation contributes to the binding to IgG1s by stabilizing the interaction to a degree that is highly dependent on the type of the *N*-glycans present.^31,40,41^ Human FcγRIIIa is glycosylated on two sites, namely, N45 and N162; see Figure 1. These two sites are very different in terms of their surrounding protein landscape; while N162 is highly exposed to the solvent, N45 is located in the core of one of the two structural domains. To understand the effect of the protein surface landscape on the oligomannoses structure and dynamics, we studied two FcγRIIIa glycoforms, one with Man5 at N45 and N162 and the other with Man9 at N45 and N162.

**Figure 7:**
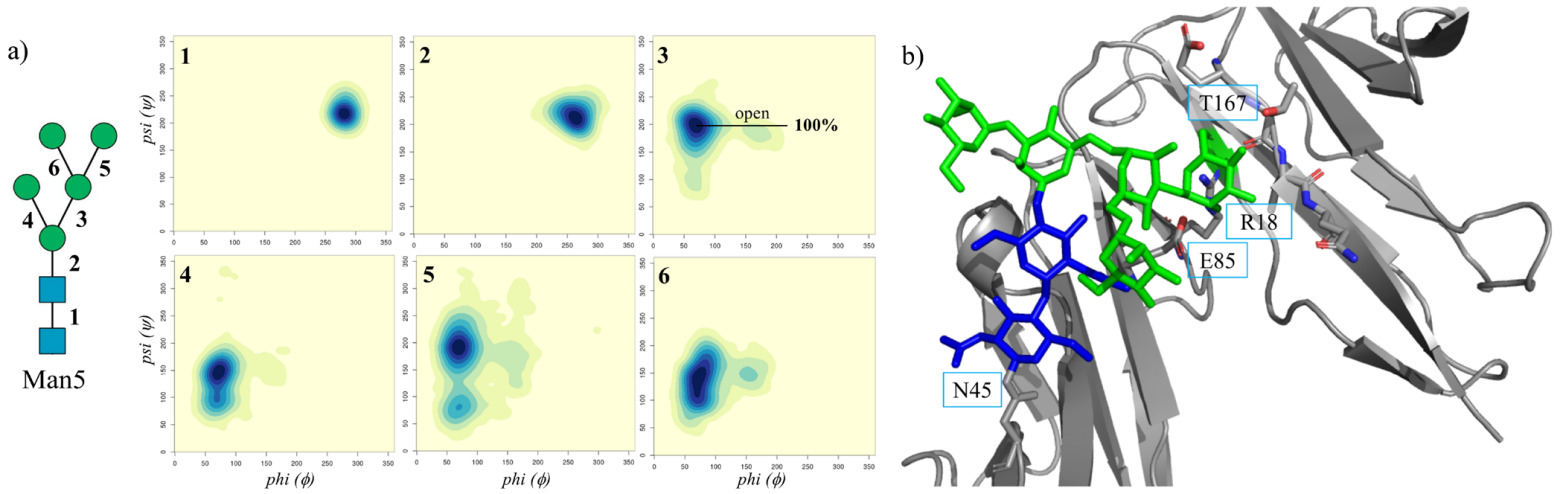
(a) Conformational analysis in terms of the ϕ and ψ torsion angles of the N40-linked Man5 linked explored during the 2 μs of cumulative MD sampling of the Man5 glycosylated FcγRIIIa. Each torsion is numbered as indicated on the left-hand side, and the corresponding heat maps are labeled in the top-left corner accordingly. (b) Dominant conformation of the N45-linked Man5, see Table 3, with the terminal Man on the (1–3) branch of the (1–6) arm restrained by hydrogen bonds to residues T167, R18 and E85, labeled in the figure. Heat maps were made with RStudio (www.rstudio.com), and structure was rendered with PyMol (www.pymol.org). *N*-glycan is colored according to the SNFG convention.

As shown in Figure 7, results obtained from 2 μs of cumulative sampling from two independent runs show that the conformational dynamics of the Man5 at N45 is significantly restrained compared to the unlinked form. Indeed, a network of hydrogen bonds connects the terminal Man on the (1–3) branch of the (1–6) arm within a protein’s cleft located between the two domains. These interactions result in shifting the Man5 intrinsic conformational equilibrium so that at N45 the Man5 (1–6) arm is mainly allowed in the open (cluster 2) conformation; see Figure 7 and Table 3. The flexibility of the (1–6) branch and of the (1–3) arm, not interacting with the protein, is the same as found for the unlinked Man5; see also Figure 2.

**Table 3:** Torsion Angle Median Values of the Linkages in the N45-Linked Man5 and Man9 (1–6) Arm^a^

Manα(1–6)-Man arm (3)	ϕ	ψ	ω	population (%)
N45-Man5				
open (cluster 2)	69 (10)	–162 (13)	–172 (11)	100
N45-Man9				
open (cluster 1)	71 (12)	174 (13)	59 (15)	57
open (cluster 3)	72 (9)	–174 (15)	–75 (11)	15
back fold (cluster 2)	67 (7)	–112 (12)	–69 (12)	28

a Standard deviation values are shown in parentheses, with relative populations obtained from clustering analysis. Angle values are in degrees. The number in parentheses in the first column indicates the linkages, as shown on the Man5 and Man9 sketches in Figures 2 and 5.

Man9 has two Manα(1–2)-Man linkages elongating both branches on the (1–6) arm, denying the pose found for Man5, which indeed disappears; see Tables 3 and S.13. Despite a higher flexibility relative to Man5, the N45-linked Man9 is less dynamic relative to the unlinked form due to the protein’s landscape. Indeed, as shown in Table 3, only three out of the seven populated conformers are accessible.

**Figure 8:**
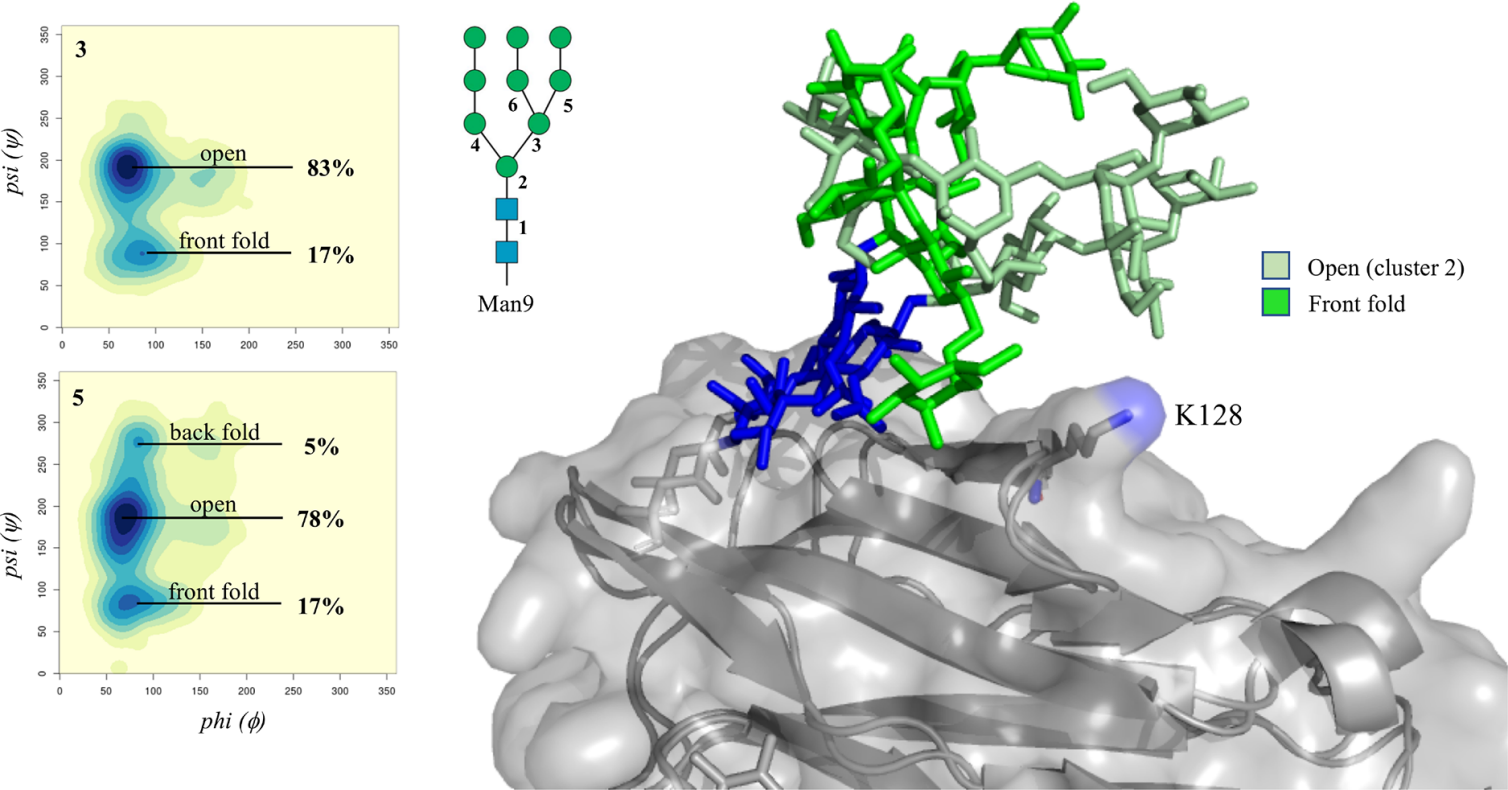
Conformational analysis of the (1–6) arm and (1–6) branch of the N162-linked Man9 in terms of ϕ and ψ torsion angles, obtained from the 2 μs of cumulative MD sampling of the Man9 glycosylated FcγRIIIa. Heat maps are labeled in the top-left corner according to the Man9 numbering in the sketch. The two dominant conformations of the N162-linked Man9 are shown on the right-hand side, with the protein represented by the solvent accessible surface and underlying cartoons in gray and the mannose residues with different shades of green as described in the legend. Heat maps were made with RStudio (www.rstudio.com), and structure was rendered with pyMol (www.pymol.org). *N*-glycans are colored according to the SNFG convention.

As shown in Figure 1, the N162 position is much more exposed to the solvent relative to N45. Consequently, the intrinsic dynamics of the N162-Man5 is almost entirely retained, with a shift promoting the open (cluster 2) relative to the open (cluster 1) as the dominant conformer; see Table 4. Meanwhile in the case of a N162-linked Man9, the dynamics of the longer arms is limited due to the proximity to the protein’s surface, see Figure 8, and in particular due to the presence of Lys 128, which because of its position denies a number of conformers due to steric hindrance and also potentially stabilizes the open (cluster 1) conformation through a hydrogen bonding interaction with the α(1–6)-linked Man on the (1–6) arm.

**Table 4:** Torsion Angle Median Values of the Linkages in the N162-Linked Man5 and Man9 (1–6) Arm^a^

Manα(1–6)-Man arm (3)	ϕ	ψ	ω	population (%)
N162-Man5				
open (cluster 2)	72 (12)	–173 (17)	–169 (15)	83
open (cluster 1)	69 (11)	–175 (16)	52 (12)	10
front fold	74 (13)	88 (13)	53 (12)	7
N162-Man9				
open (cluster 1)	70 (11)	–171 (16)	55 (11)	83
front fold	83 (14)	88 (12)	48 (9)	17

a Standard deviation values are shown in parentheses, with relative populations obtained from clustering analysis. Angle values are in degrees. The number in parentheses in the first column indicates the linkages, as shown on the Man5 and Man9 sketches in Figures 2 and 5.

## Discussion

In this work we analyzed the 3D structure and dynamics of human oligomannose *N*-glycans, from Man5 to Man9, when free (unlinked) in solution and also determined how the effect of FcγRIIIa (CD16a) surface landscape modulates their structural equilibria. Despite similarities with complex *N*-glycans,^16,17^ in terms of the core chitobiose rigidity and of the relatively low degree of flexibility of the (1–3) arm, oligomannoses have a very unique architecture, which changes with the progressive functionalization of the arms. More specifically, Man5 shows a clear propensity for an “open” structure, where the (1–6) arm is outstretched orientating the two branches on either side of the (1–3) arm; see Figures 2 and 3. Small variations of the open structure, determined by two accessible values of the (1–6) arm ω torsion, are also populated, see Figure 3, and of the additional degrees of freedom of both (1–3/6) branches, which closely reflect the dynamics of the arms, see Table 1. In larger oligomannoses the (1–3) arm and (1–3/6) branches on the (1–6) arm are terminated with Manα(1–2)-Man groups, giving raise to different Man6 up to Man8 isoforms and then to Man9. We analyzed 10 different positional isomers of Man6 to Man8, see Figure S.1, and focused our attention on the isoforms named (I) as representative examples, shown in Figure 1, while including all others for completeness as Supporting Information. Sampling results show that the Manα(1–2)-Man linkages are rigid and do not significantly affect the intrinsic dynamics of the other linkages. The most interesting and unique aspect of the arms elongation in oligomannoses is that the orientation of the additional Manα(1–2)-Man linkages within the underlying architecture of Man5 determines a progressive increase of interarm contacts, see Figure 6; so the structure of Man9 is quite compact, or more “tree-like”, relative to smaller oligomannoses, where the arms are shorter but characterized by a more independent dynamics. As a further step in the analysis, a direct comparison of the results we obtained for Man9 and Man8(II) with NMR-validated REMD analysis^42^ shows a very good agreement, supporting that the trimming of terminal residues allows for more extended arm structures, which expose embedded glycotopes; see Figure S.18.

The results obtained for the unlinked oligomannoses also confirm an earlier observation we made in the context of complex *N*-glycans,^16^ whereby the overall 3D architecture is determined by the local spatial arrangement of independent groups of monosaccharides we named “glycoblocks”. The oligomannoses dynamics can be also discretized in terms of these structural units,^16^ with the addition of a unique Manα(1–2)-Man glycoblock that can be added to the arms with, as we have seen, minimal effect to the dynamics of the underlying units it builds on. This observation can offer a practical advantage to the study of glycan recognition through molecular docking, for example, where the receptor binds a specific glycoblock unit and recognition depends only on its accessibility within a specific glycoform.

To understand how the protein affects the presentation of the glycans to potential receptors, we have looked at the human FcγRIIIa (CD16a). Human FcγRIIIa has two N-glycosylation sites, namely, N45 and N162, where the type of glycosylation affects the receptor’s binding affinity to IgG1s.^31,32,43^ The surface landscape around these two sites is quite different, with N162 exposed to the solvent while N45 is located in the core of one of the two structural domains; see Figure 1. Conformational sampling of a Man5 at N45 shows that the (1–6) arm dynamics is heavily restrained to one of its two open conformations accessible in solution; see Figure 7. More specifically, we found that the terminal Man on the (1–3) branch is engaged in a network of hydrogen bonding interactions involving a number of residues near the glycosylation site, namely, Arg 18, Glu 85, and Thr 167. The stabilization of this glycoform by the FcγRIIIa surface landscape renders the (1–3) branch on the (1–6) arm virtually inaccessible for further functionalization. This result agrees with recent work highlighting the unique prevalence of hybrid and oligomannose type *N*-glycans at N45.^32,43^ The N162 position determines very little steric hindrance to the dynamics of Man5, which retains most of the degrees of freedom characterized for the glycan free in solution. Meanwhile, the dynamics of the larger Man9 is greatly affected by the presence of Lys 128, which forces the glycan to adopt only two of the conformations accessible to the unlinked form; see Table 4 and Figure 8. Ultimately, the comparison between the conformational propensity of the unlinked Man5 and Man9 oligomannoses relative to their FcγRIIIa-linked counterparts suggests that the protein landscape affects the glycans structure by shifting their intrinsic conformational equilibria toward forms that complement it, yet it does not actively morph the glycan into unnatural conformers.

## Conclusions

In this work we have characterized the 3D structure and dynamics of human oligomannose *N*-glycans unlinked and linked to FcγRIIIa through extensive sampling based on conventional MD simulations. The simulations of the unlinked oligomannose *N*-glycans show a complex architecture that is derived from a progressively intricate network of transient hydrogen bonding interactions involving the terminal residues on the arms, all linked through rigid Manα(1–2)-Man glycoblocks. The protein landscape affects the conformational equilibrium of the *N*-glycans favoring conformations that complement it, but it does not actively distort the oligomannoses’ structure. Indeed, the two FcγRIIIa glycosylation sites studied in this work present different sets of constraints to different glycoforms and accordingly shift each conformational equilibrium specifically. This determines a diverse degree of accessibility of the arms for further functionalization by glycotransferases and glycohydrolases at N45,^32,43^ which has been found to have an unusually high degree of hybrid *N*-glycoforms, and ultimately exposure of the arms at N162 for contact with the IgG1 Fc *N*-glycans, which is implicated in modulating ADCC.^19,31,44,45^ Work in this direction is currently ongoing in our lab.

## Supporting Information Available

Seventeen tables and eighteen figures providing a complete list of all rotamers conformations with relative populations for all oligomannoses isomers studied in this work, with Figure S.18 showing additional data analysis (PDF)

## Notes

**Electronic Data Sharing.** All structures and trajectories will be made available through a database currently under development in our lab. In the meantime, distribution is done based on requests to the corresponding author.
