# Supplementary material for: Oligomannose N-Glycans 3D Architecture and Its Response to the FcγRIIIa Structural Landscape
Source: J Phys Chem B. 2021 Mar 4;125(10):2607–16. doi: 10.1021/acs.jpcb.1c00304 (PMC8279474; doi:10.1021/acs.jpcb.1c00304)
Supplement: Supplementary file 1 — jp1c00304_si_001.pdf [file jp1c00304_si_001.pdf]

Supporting Information for Publication The Oligomannose N-glycans 3D Architecture and its Response to the FcγRIIIa Structural Landscape

Carl Aaron Fogarty[1], Elisa Fadda[1]

[1]Department of Chemistry, Maynooth University, Ireland.

February 22, 2021

Contents

1 Oligomannose Isomers 1

2 Man 5 2

3 Man 6 I 4

4 Man 6 II 6

5 Man 6 III 8

6 Man 7 I 10

7 Man 7 II 12

8 Man 7 III 14

9 Man 7 IV 16

10 Man 8 I 18

11 Man 8 II 20

12 Man 8 III 22

13 Man 9 24

14 FcγRC: Man5 N45 26

15 FcγRC: Man5 N162 28

16 FcγRC: Man9 N45 30

17 FcγRC: Man9 N162 32

18 DBSCAN Parameters 34

19 Man 9 / 8(II) Distance Measurements 35

1 Oligomannose Isomers

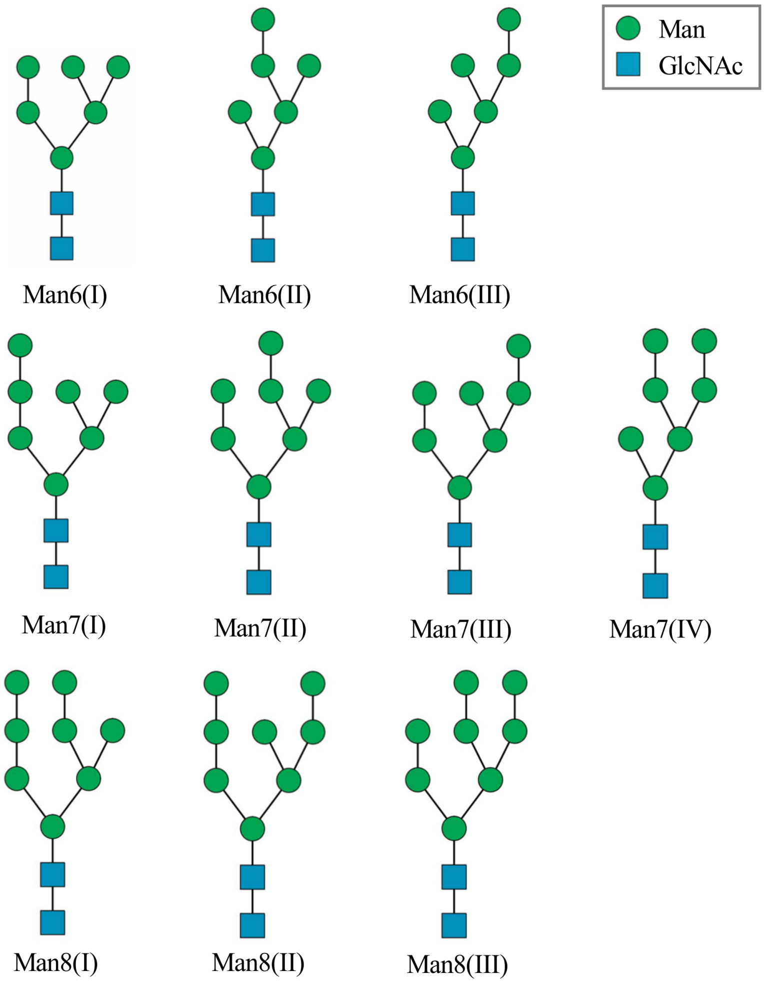

Figure S. 1: SNFG representation of all the Man6-8 oligomannose positional isomers studied in this work in addition to Man5/9

2 Man 5

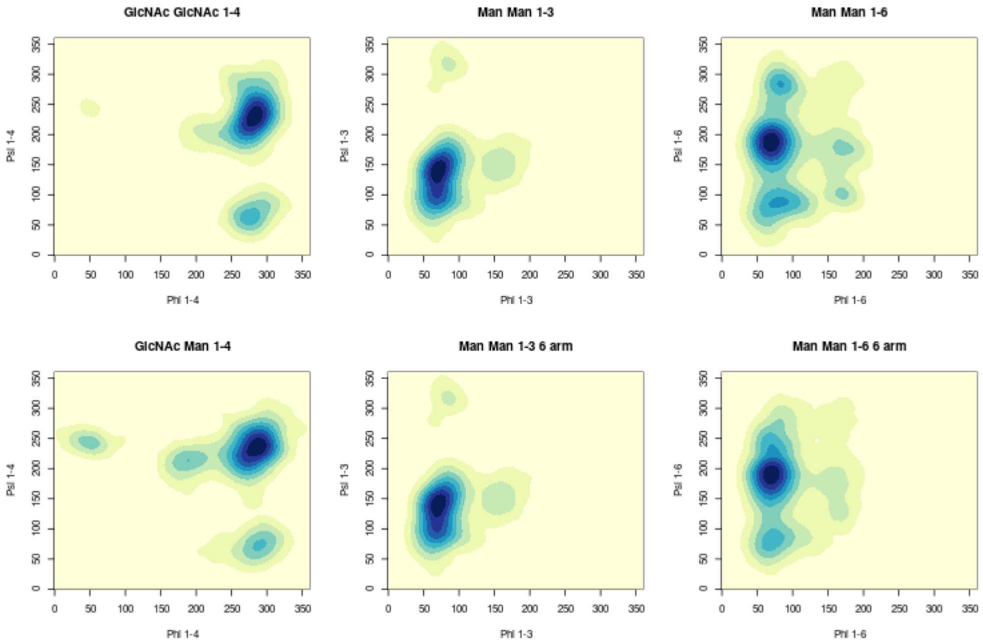

Figure S. 2: 2D Kernel density estimates for the ϕ / ψ angle distributions for Man 5.

Table S. 1: The ϕ / ψ / ω angle distributions for Man 5.

**GlcNAc β(1-4) GlcNAc**	ϕ	ψ	ω	**Pop (%)**
Cluster 1	-78.1 (11.2)	-130.2 (18.3)	-	94.6
Cluster 2	-81.9 (11.4)	65.5 (11.2)	-	5.4
**Man β(1-4) GlcNAc**	ϕ	ψ	ω	**Pop (%)**
Cluster 1	-76.0 (12.9)	-125.9 (15.6)	-	93.0
Cluster 2	-70.5 (10.7)	72.9 (10.9)	-	3.5
Cluster 3	-170.7 (12.3)	-146.8 (7.83)	-	2.5
Cluster 4	48.6 (12.9)	-116.3 (5.5)	-	1.0
**Man α(1-3) Man (1-3)**	ϕ	ψ	ω	**Pop (%)**
Cluster 1	71.7 (8.1)	141.6 (14.5)	-	73.9
Cluster 2	68.8 (9.4)	99.7 (10.5)	-	26.1
**Man α(1-3) Man (1-6)**	ϕ	ψ	ω	**Pop (%)**
Cluster 1	72.1 (9.3)	138.6 (15.1)	-	62.5
Cluster 2	67.6 (9.9)	99.9 (10.4)	-	37.5
**Man α(1-6) Man**	ϕ	ψ	ω	**Pop (%)**
Cluster 1	71.1 (10.7)	-172.5 (17.0)	56.1 (10.8)	48.6
Cluster 2	67.8 (10.0)	-175.4 (14.0)	-175.4 (12.7)	33.3
Cluster 3	79.2 (16.4)	86.7 (13.4)	50.9 (10.0)	12.4
Cluster 4	82.6 (8.6)	-76.5 (10.8)	-150.2 (10.5)	5.7
**Man α(1-6) Man (1-6)**	ϕ	ψ	ω	**Pop (%)**
Cluster 1	70.3 (10.3)	-171.2 (15.9)	54.8 (10.5)	80.4
Cluster 2	69.6 (8.5)	-173.3 (13.4)	-80.7 (12.6)	5.7
Cluster 3	69.6 (7.6)	-120.2 (13.6)	-64.3 (10.5)	5.6
Cluster 4	69.7 (7.6)	-168.7 (17.5)	-164.4(9.4)	4.4
Cluster 5	70.9 (10.5)	82.9 (11.6)	47.9 (8.8)	3.9

3 Man 6 I

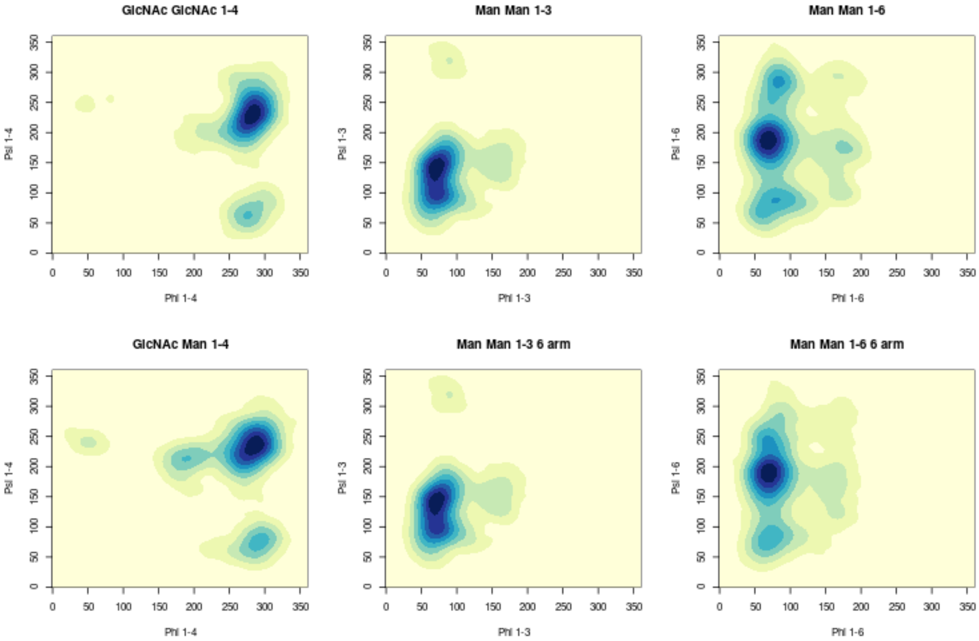

Figure S. 3: 2D Kernel density estimates for the ϕ / ψ angle distributions for Man 6 I.

Table S. 2: The ϕ / ψ / ω angle distributions for Man 6 I.

**GlcNAc β(1-4) GlcNAc**	ϕ	ψ	ω	**Pop (%)**
Cluster 1	-78.0 (10.9)	-130.3 (17.4)	-	96.7
Cluster 2	-82.5 (10.7)	64.7 (11.1)	-	3.3
**Man β(1-4) GlcNAc**	ϕ	ψ	ω	**Pop (%)**
Cluster 1	-76.2 (12.9)	-126.4 (15.4)	-	92.4
Cluster 2	-68.5 (10.5)	74.4 (10.94)	-	4.8
Cluster 3	-169.1 (12.1)	-146.2 (8.3)	-	2.8
**Man α(1-3) Man (1-3)**	ϕ	ψ	ω	**Pop (%)**
Cluster 1	72.1 (9.1)	142.1 (14.4)	-	67.8
Cluster 2	71.1 (9.9)	98.5 (10.4)	-	32.2
**Man α(1-3) Man (1-6)**	ϕ	ψ	ω	**Pop (%)**
Cluster 1	72.3 (9.2)	138.6 (15.1)	-	62.2
Cluster 2	67.7 (9.7)	99.7 (10.3)	-	37.8
**Man α(1-6) Man**	ϕ	ψ	ω	**Pop (%)**
Cluster 1	71.0 (10.7)	-172.2 (15.8)	55.9 (10.8)	41.1
Cluster 2	67.7 (9.9)	-175.1 (14.1)	-175.8 (12.3)	37.5
Cluster 3	76.6 (16.5)	85.1 (14.6)	50.3 (9.9)	10.9
Cluster 4	83.3 (8.5)	-75.3 (11.7)	-149.0 (10.8)	7.1
Cluster 5	70.7 (8.7)	-177.6 (13.6)	-71.9 (10.1)	3.4
**Man α(1-6) Man (1-6)**	ϕ	ψ	ω	**Pop (%)**
Cluster 1	70.2 (10.4)	-171.3 (15.7)	54.7 (10.3)	76.0
Cluster 2	69.2 (8.7)	-173.9 (13.6)	-82.4 (13.3)	7.8
Cluster 3	68.7 (7.8)	-119.6 (14.1)	-65.1 (10.8)	8.5
Cluster 4	70.2 (7.8)	-175.5 (17.5)	-163.6 (8.9)	4.4
Cluster 5	71.3 (9.3)	82.0 (10.1)	47.5 (8.0)	3.2
**Man α(1-2) Man (1-3)**	ϕ	ψ	ω	**Pop (%)**
Cluster 1	74.3 (8.7)	150.7 (15.1)	-	72.7
Cluster 2	70.0 (9.4)	107.2 (11.6)	-	27.3

4 Man 6 II

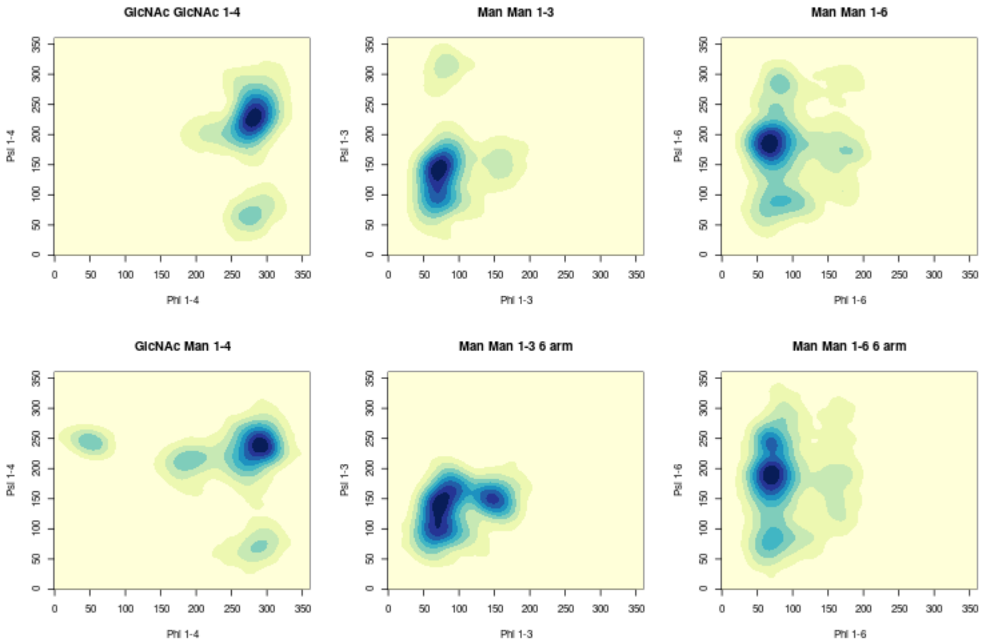

Figure S. 4: 2D Kernel density estimates for the ϕ / ψ angle distributions for Man 6 II.

Table S. 3: The ϕ / ψ / ω angle distributions for Man 6 II.

**GlcNAc β(1-4) GlcNAc**	ϕ	ψ	ω	**Pop (%)**
Cluster 1	-78.9 (10.3)	-131.7 (16.7)	-	98.0
Cluster 2	-83.0 (9.4)	65.8 (9.0)	-	2.0
**Man β(1-4) GlcNAc**	ϕ	ψ	ω	**Pop (%)**
Cluster 1	-71.5 (11.8)	-122.8 (13.8)	-	94.2
Cluster 2	-70.3 (7.8)	70.7 (6.5)	-	1.0
Cluster 3	-170.2 (12.0)	-146.2 (8.0)	-	2.8
Cluster 4	50.7 (8.2)	-116.0 (5.7)	-	2.0
**Man α(1-3) Man (1-3)**	ϕ	ψ	ω	**Pop (%)**
Cluster 1	72.3 (9.2)	138.6 (15.1)	-	62.2
Cluster 2	67.7 (9.7)	99.7 (10.3)	-	37.8
**Man α(1-3) Man (1-6)**	ϕ	ψ	ω	**Pop (%)**
Cluster 1	76.1 (11.3)	144.6 (16.6)	-	57.0
Cluster 2	70.3 (10.5)	100.3 (9.5)	-	24.8
Cluster 3	147.4 (10.5)	150.2 (9.6)	-	18.3
**Man α(1-6) Man**	ϕ	ψ	ω	**Pop (%)**
Cluster 1	71.0 (10.7)	-172.2 (15.8)	56.2 (11.4)	58.2
Cluster 2	65.4 (16.3)	-178.9 (11.8)	-177.4 (11.2)	33.9
Cluster 3	82.1 (16.4)	89.2 (13.7)	51.8 (9.9)	6.3
Cluster 4	81.7 (7.4)	-75.6 (9.1)	-149.2 (9.2)	1.6
**Man α(1-6) Man (1-6)**	ϕ	ψ	ω	**Pop (%)**
Cluster 1	70.3 (10.5)	-171.4 (16.2)	54.7 (10.6)	70.28
Cluster 2	71.3(7.1)	179.1 (10.22)	-72.0 (8.3)	3.9
Cluster 3	69.5 (7.2)	-119.1 (14.0)	-67.1 (9.5)	14.5
Cluster 4	70.7 (9.7)	-173.3 (23.8)	-164.6 (13.2)	6.9
Cluster 5	71.5 (12.0)	84.2 (13.6)	49.0 (10.2)	4.3
**Man α(1-2) Man (1-3)**	ϕ	ψ	ω	**Pop (%)**
Cluster 1	72.3 (8.0)	149.3 (13.4)	-	74.8
Cluster 2	69.6 (7.9)	112.5 (10.1)	-	25.2

5 Man 6 III

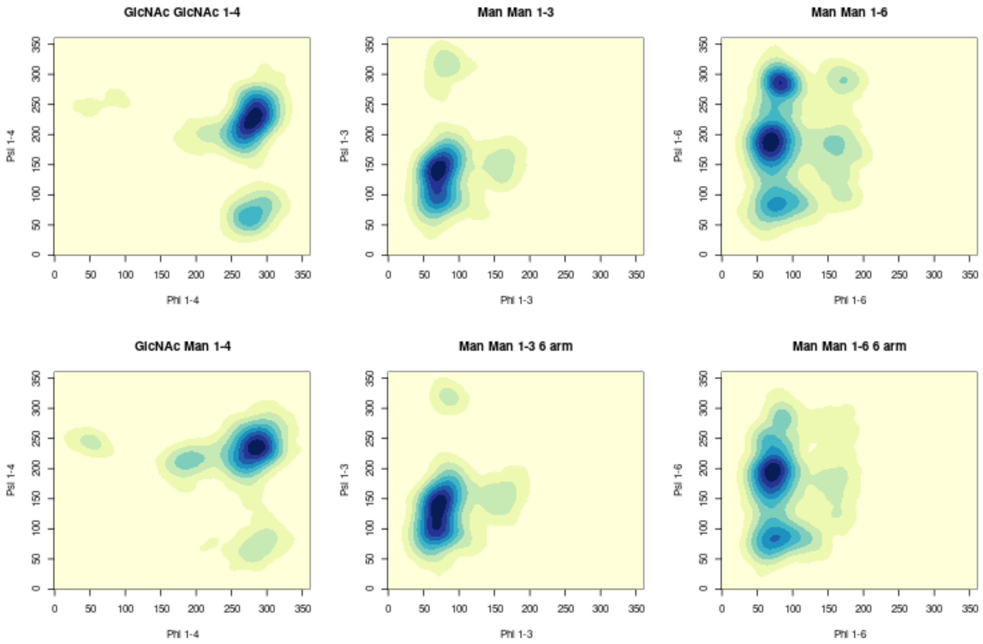

Figure S. 5: 2D Kernel density estimates for the ϕ / ψ angle distributions for Man 6 III.

Table S. 4: The ϕ / ψ / ω angle distributions for Man 6 III.

**GlcNAc β(1-4) GlcNAc**	ϕ	ψ	ω	**Pop (%)**
Cluster 1	-78.8 (11.4)	-133.8 (17.5)	-	93.5
Cluster 2	-82.0 (11.8)	66.0 (11.7)	-	6.5
**Man β(1-4) GlcNAc**	ϕ	ψ	ω	**Pop (%)**
Cluster 1	- 76.3 (12.6)	-125.5 (14.4)	-	97.9
Cluster 2	-170.4 (10.9)	-146.2 (7.5)	-	2.1
**Man α(1-3) Man (1-3)**	ϕ	ψ	ω	**Pop (%)**
Cluster 1	76.1 (11.3)	144.6 (16.6)	-	57.0
Cluster 2	70.3 (10.5)	100.3 (9.5)	-	24.8
**Man α(1-3) Man (1-6)**	ϕ	ψ	ω	**Pop (%)**
Cluster 1	72.3 (9.2)	138.6 (15.1)	-	62.2
Cluster 2	67.7 (9.7)	99.7 (10.3)	-	37.8
**Man α(1-6) Man**	ϕ	ψ	ω	**Pop (%)**
Cluster 1	71.2 (10.7)	-172.9 (17.2)	56.0 (10.7)	36.1
Cluster 2	67.5 (10.7)	-174.3 (15.2)	-175.9 (12.4)	28.6
Cluster 3	79.1 (14.8)	86.4 (12.9)	50.1 (10.7)	9.7
Cluster 4	82.3 (7.7)	-74.5 (9.8)	-151.5 (9.7)	24.2
Cluster 5	71.6 (6.9)	-173.2 (11.5)	-69.2 (8.3)	1.4
**Man α(1-6) Man (1-6)**	ϕ	ψ	ω	**Pop (%)**
Cluster 1	71.9 (10.2)	-166.8 (16.7)	55.1 (10.1)	68.2
Cluster 2	69.2 (8.2)	-173.8 (12.6)	- 83.8 (11.2)	4.0
Cluster 3	68.9 (7.6)	-120.6 (11.9)	- 65.4 (10.0)	4.0
Cluster 4	69.0 (8.9)	-175.7 (23.2)	-165.9 (10.6)	4.5
Cluster 5	78.2 (16.7)	86.2 (10.1)	49.8 (10.1)	12.3
Cluster 6	71.9 (10.4)	82.6 (12.5)	-166.3 (17.7)	6.1
Cluster 7	84.9 (5.7)	- 78.2 (7.5)	- 79.57 (6.4)	1.0
**Man α(1-2) Man (1-6)(1-6)**	ϕ	ψ	ω	**Pop (%)**
Cluster 1	74.7 (8.6)	152.1 (15.1)	-	79.1
Cluster 2	71.3 (9.6)	106.4 (12.2)	-	20.9

6 Man 7 I

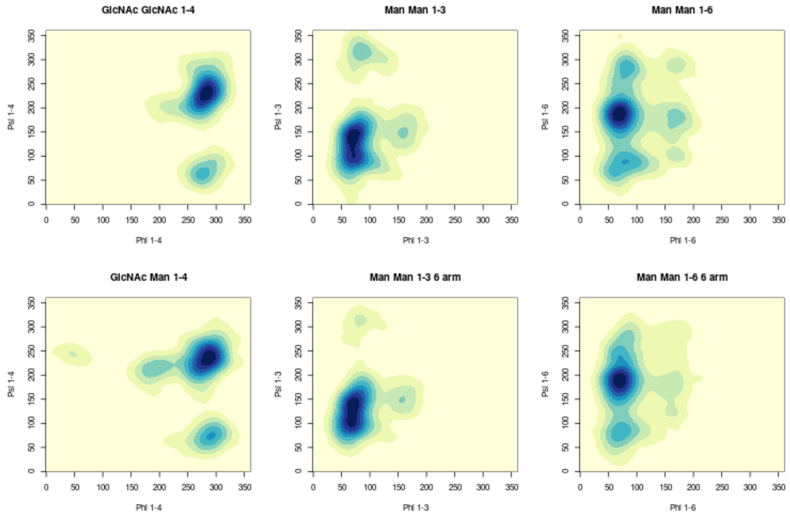

Figure S. 6: 2D Kernel density estimates for the ϕ / ψ angle distributions for Man 7 I.

Table S. 5: The ϕ / ψ / ω angle distributions for Man 7 I.

**GlcNAc β(1-4) GlcNAc**	ϕ	ψ	ω	**Pop (%)**
Cluster 1	-77.9 (10.9)	-130.2 (17.5)	-	95.2
Cluster 2	-82.1 (10.8)	65.5 (11.4)	-	4.8
**Man β(1-4) GlcNAc**	ϕ	ψ	ω	**Pop (%)**
Cluster 1	-76.6 (12.8)	-125.5 (15.4)	-	89.8
Cluster 2	-69.3 (10.9)	73.5 (11.3)	-	7.7
Cluster 3	-167.7 (12.2)	-145.2 (8.3)	-	2.5
**Man α(1-3) Man (1-3)**	ϕ	ψ	ω	**Pop (%)**
Cluster 1	72.1 (9.0)	141.5 (14.1)	-	63.3
Cluster 2	71.3 (10.3)	97.6 (10.7)	-	36.7
**Man α(1-3) Man (1-6)**	ϕ	ψ	ω	**Pop (%)**
Cluster 1	71.9 (9.2)	138.6 (14.8)	-	61.1
Cluster 2	67.5 (9.9)	99.5 (10.4)	-	38.9
**Man α(1-6) Man**	ϕ	ψ	ω	**Pop (%)**
Cluster 1	70.7 (10.8)	-172.3 (15.8)	55.4 (11.2)	46.2
Cluster 2	68.0 (10.0)	-175.6 (14.1)	-175.6 (12.3)	33.1
Cluster 3	76.6 (16.5)	84.9 (14.7)	49.8 (9.8)	11.7
Cluster 4	82.0 (8.3)	-76.0 (10.9)	-148.8 (10.6)	4.6
Cluster 5	71.5 (9.1)	-178.9 (14.6)	-71.8 (11.5)	4.4
**Man α(1-6) Man (1-6)**	ϕ	ψ	ω	**Pop (%)**
Cluster 1	70.2 (10.4)	-171.5 (15.4)	54.4 (10.4)	78.1
Cluster 2	69.1 (8.3)	-175.9 (12.8)	-79.3 (12.2)	5.7
Cluster 3	69.2 (7.8)	-118.2 (13.2)	-65.1 (10.1)	5.4
Cluster 4	69.8 (8.5)	-172.0 (18.5)	-165.6 (10.0)	7.4
Cluster 5	71.3 (9.5)	84.0 (10.3)	47.7 (8.0)	3.4
**Man α(1-2) Man (1-3)**	ϕ	ψ	ω	**Pop (%)**
Cluster 1	74.6 (9.0)	152.5 (15.4)	-	72.0
Cluster 2	70.0 (9.7)	105.3 (11.6)	-	28.0
**Man α(1-2) Man (1-3)**	ϕ	ψ	ω	**Pop (%)**
Cluster 1	74.1 (8.8)	151.4 (15.0)	-	76.0
Cluster 2	70.0 (9.3)	106.5 (11.6)	-	24.0

7 Man 7 II

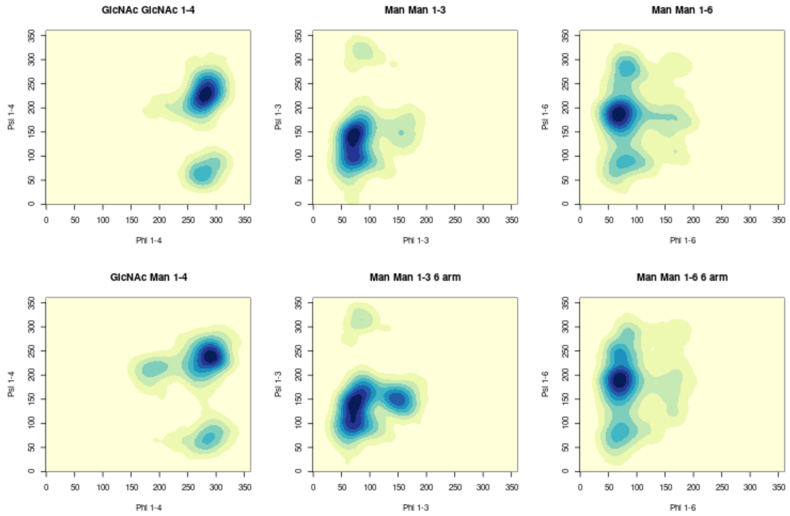

Figure S. 7: 2D Kernel density estimates for the ϕ / ψ angle distributions for Man 7 II.

Table S. 6: The ϕ / ψ / ω angle distributions for Man 7 II.

**GlcNAc β(1-4) GlcNAc**	ϕ	ψ	ω	**Pop (%)**
Cluster 1	-78.8 (10.2)	-131.6 (16.0)	-	92.8
Cluster 2	-81.8 (11.8)	65.5 (11.4)	-	7.2
**Man β(1-4) GlcNAc**	ϕ	ψ	ω	**Pop (%)**
Cluster 1	-71.7 (12.8)	-125.5 (15.4)	-	92.3
Cluster 2	-72.5 (11.4)	69.3 (11.6)	-	5.0
Cluster 3	-170.2 (12.3)	-146.7 (8.0)	-	2.7
**Man α(1-3) Man (1-3)**	ϕ	ψ	ω	**Pop (%)**
Cluster 1	72.5 (9.2)	142.0 (14.1)	-	67.6
Cluster 2	70.8 (10.2)	98.2 (10.5)	-	32.4
**Man α(1-3) Man (1-6)**	ϕ	ψ	ω	**Pop (%)**
Cluster 1	75.9 (11.1)	138.6 (14.8)	-	59.0
Cluster 2	70.4 (10.7)	100.2 (9.6)	-	26.5
Cluster 3	148.9 (10.2)	150.6 (9.7)	-	14.5
**Man α(1-6) Man**	ϕ	ψ	ω	**Pop (%)**
Cluster 1	71.1 (12.5)	-171.9 (15.6)	56.1 (11.9)	55.5
Cluster 2	66.7 (10.2)	-177.4 (13.0)	-175.7 (12.4)	31.7
Cluster 3	79.6 (15.6)	87.9 (13.3)	50.1 (9.7)	6.5
Cluster 4	82.4 (8.8)	- 77.6 (10.8)	-150.0 (10.3)	5.2
Cluster 5	71.6 (7.2)	-177.4 (9.8)	- 71.7 (7.9)	1.2
**Man α(1-6) Man (1-6)**	ϕ	ψ	ω	**Pop (%)**
Cluster 1	70.3 (10.5)	-170.8 (16.1)	54.8 (10.5)	71.9
Cluster 2	69.8 (7.8)	-179.8 (14.0)	- 74.4 (9.6)	5.0
Cluster 3	70.0 (7.5)	-117.8 (13.4)	- 67.3 (10.1)	8.5
Cluster 4	70.7 (9.8)	-172.4 (24.5)	-163.9 (12.9)	8.3
Cluster 5	71.0 (12.2)	84.4 (13.6)	48.8 (9.9)	4.4
Cluster 6	67.8 (10.7)	73.6 (12.5)	-177.1 (10.1)	1.9
**Man α(1-2) Man (1-3)**	ϕ	ψ	ω	**Pop (%)**
Cluster 1	74.2 (8.7)	150.6 (14.7)	-	72.5
Cluster 2	70.0 (9.2)	107.4 (11.6)	-	27.5
**Man α(1-2) Man (1-3)(1-3)**	ϕ	ψ	ω	**Pop (%)**
Cluster 1	72.4 (8.1)	149.5 (13.6)	-	75.9
Cluster 2	69.7 (8.2)	111.7 (10.6)	-	24.1

8 Man 7 III

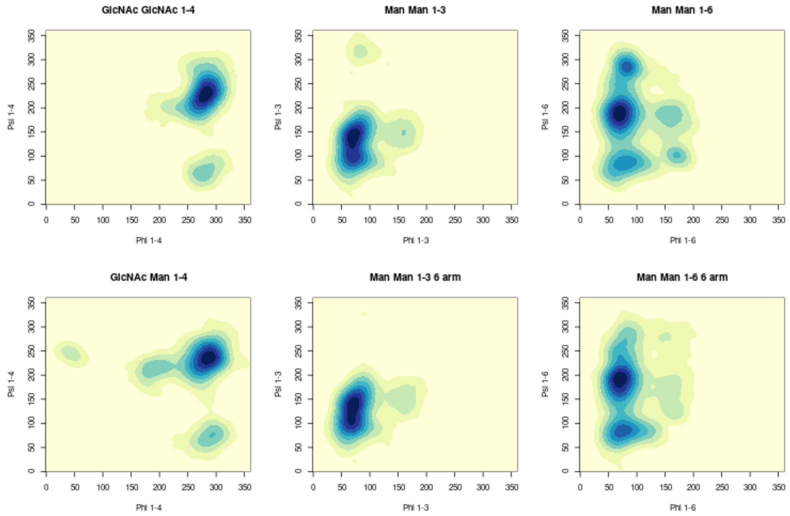

Figure S. 8: 2D Kernel density estimates for the ϕ / ψ angle distributions for Man 7 III.

Table S. 7: The ϕ / ψ / ω angle distributions for Man 7 III.

**GlcNAc β(1-4) GlcNAc**	ϕ	ψ	ω	**Pop (%)**
Cluster 1	-78.9 (11.5)	-131.9 (18.3)	-	97.7
Cluster 2	-82.7 (9.8)	65.3 (9.0)	-	2.3
**Man β(1-4) GlcNAc**	ϕ	ψ	ω	**Pop (%)**
Cluster 1	-75.4 (12.7)	-125.3 (14.8)	-	94.0
Cluster 2	-68.4 (10.4)	73.5 (10.4)	-	3.1
Cluster 3	-167.5 (12.4)	-145.6 (8.0)	-	2.9
**Man α(1-3) Man (1-3)**	ϕ	ψ	ω	**Pop (%)**
Cluster 1	72.1 (9.0)	141.5 (14.5)	-	66.5
Cluster 2	70.4 (9.9)	98.3 (10.4)	-	33.5
**Man α(1-3) Man (1-6)**	ϕ	ψ	ω	**Pop (%)**
Cluster 1	71.8 (9.1)	139.1 (14.6)	-	65.1
Cluster 2	67.8 (9.5)	100.4 (9.8)	-	34.9
**Man α(1-6) Man**	ϕ	ψ	ω	**Pop (%)**
Cluster 1	71.1 (10.7)	-172.4 (16.1)	56.0 (10.7)	42.6
Cluster 2	67.2 (9.4)	-175.5 (15.4)	-176.2 (11.7)	20.2
Cluster 3	78.0 (16.2)	85.3 (13.5)	49.5 (10.3)	14.5
Cluster 4	82.4 (7.7)	- 74.3 (9.9)	-151.0 (9.4)	11.3
Cluster 5	73.3 (9.5)	-160.8 (20.5)	- 66.1 (12.0)	7.8
Cluster 6	72.8 (8.6)	- 98.6 (10.0)	- 73.0 (9.1)	1.9
Cluster 7	169.8 (7.8)	102.0 (7.2)	171.2 (7.6)	1.7
**Man α(1-6) Man (1-6)**	ϕ	ψ	ω	**Pop (%)**
Cluster 1	71.3 (10.3)	-169.3 (16.5)	55.0 (10.3)	56.7
Cluster 2	69.2 (9.1)	-174.3 (15.0)	- 79.7 (13.6)	6.2
Cluster 3	71.2 (8.9)	-113.5 (17.1)	- 67.5 (11.4)	5.0
Cluster 4	69.4 (9.6)	-178.5 (21.7)	-166.2 (12.1)	9.2
Cluster 5	79.6 (17.5)	85.8 (11.4)	49.8 (9.6)	14.5
Cluster 6	71.9 (11.1)	80.9 (11.2)	-168.0 (14.6)	8.4
**Man α(1-2) Man (1-3)**	ϕ	ψ	ω	**Pop (%)**
Cluster 1	74.2 (8.7)	150.4 (14.8)	-	72.1
Cluster 2	69.8 (9.1)	107.5 (11.3)	-	27.9
**Man α(1-2) Man (1-6)(1-6)**	ϕ	ψ	ω	**Pop (%)**
Cluster 1	74.5 (8.5)	151.5 (14.5)	-	78.0
Cluster 2	70.9 (9.1)	106.7 (11.8)	-	22.0

9 Man 7 IV

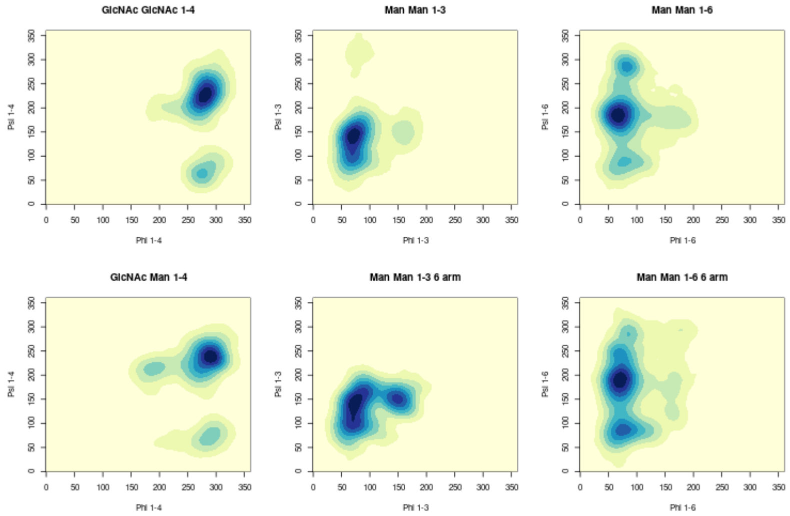

Figure S. 9: 2D Kernel density estimates for the ϕ / ψ angle distributions for Man 7 IV.

Table S. 8: The ϕ / ψ / ω angle distributions for Man 7 IV.

**GlcNAc β(1-4) GlcNAc**	ϕ	ψ	ω	**Pop (%)**
Cluster 1	-79.8 (10.2)	-133.1 (16.5)	-	95.8
Cluster 2	-82.3 (10.6)	65.8 (11.8)	-	4.2
**Man β(1-4) GlcNAc**	ϕ	ψ	ω	**Pop (%)**
Cluster 1	-71.8 (12.0)	-122.9 (14.1)	-	95.3
Cluster 2	-70.0 (10.7)	72.3 (10.9)	-	2.6
Cluster 3	-169.2 (10.7)	-145.3 (7.7	-	2.1
**Man α(1-3) Man (1-3)**	ϕ	ψ	ω	**Pop (%)**
Cluster 1	72.9 (9.1)	142.4 (14.1)	-	77.3
Cluster 2	69.3 (9.1)	100.1 (10.3)	-	22.7
**Man α(1-3) Man (1-6)**	ϕ	ψ	ω	**Pop (%)**
Cluster 1	76.6 (11.8)	145.7 (16.9)	-	56.9
Cluster 2	70.6 (10.7)	100.0 (9.7)	-	23.7
Cluster 3	148.4 (10.7)	151.5 (10.3)	-	19.4
**Man α(1-6) Man**	ϕ	ψ	ω	**Pop (%)**
Cluster 1	70.5 (12.4)	-172.4 (15.5)	55.7 (11.5)	46.0
Cluster 2	66.3 (9.9)	-178.1 (13.4)	-176.7 (11.7)	40.8
Cluster 3	78.4 (14.7)	87.6 (12.9)	50.3 (9.7)	5.1
Cluster 4	82.0 (7.5)	- 74.7 (10.1)	-151.1 (9.8)	8.1
**Man α(1-6) Man (1-6)**	ϕ	ψ	ω	**Pop (%)**
Cluster 1	71.1 (10.3)	-170.2 (16.6)	54.9 (10.4)	61.8
Cluster 2	70.2 (7.3)	177.6 (10.8)	- 72.6 (8.8)	3.0
Cluster 3	70.1 (7.5)	-118.6 (12.0)	- 66.9 (9.8)	7.5
Cluster 4	69.9 (9.2)	-179.5 (22.1)	-165.8 (11.2)	5.4
Cluster 5	79.8 (16.5)	86.1 (11.9)	50.5 (9.5)	12.5
Cluster 6	71.6 (10.2)	85.4 (12.6)	-158.7 (21.5)	8.8
Cluster 7	84.9 (5.7)	- 76.2 (7.5)	- 80.6 (6.1)	1.2
**Man α(1-2) Man (1-3)**	ϕ	ψ	ω	**Pop (%)**
Cluster 1	74.5 (8.8)	151.5 (14.9)	-	77.2
Cluster 2	70.6 (9.3)	106.2 (12.2)	-	22.8
**Man α(1-2) Man (1-6)(1-6)**	ϕ	ψ	ω	**Pop (%)**
Cluster 1	72.2 (8.0)	149.4 (13.6)	-	74.1
Cluster 2	69.7 (8.1)	112.1 (10.4)	-	25.9

10 Man 8 I

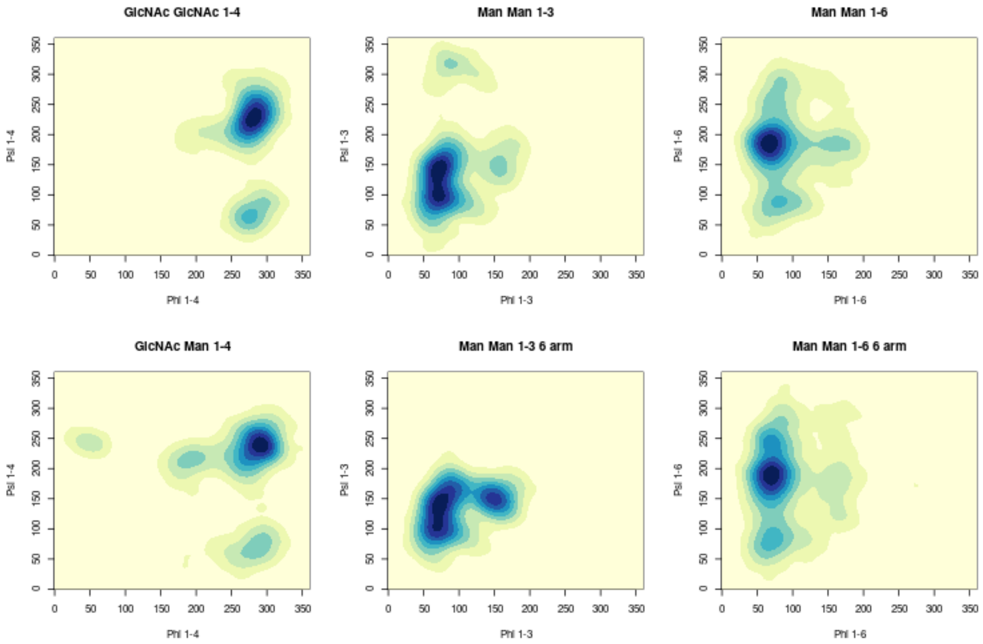

Figure S. 10: 2D Kernel density estimates for the ϕ / ψ angle distributions for Man 8 I.

Table S. 9: The ϕ / ψ / ω angle distributions for Man 8 I

**GlcNAc β(1-4) GlcNAc**	ϕ	ψ	ω	**Pop (%)**
Cluster 1	-78.8 (10.1)	-131.7 (16.2)	-	94.9
Cluster 2	-82.2 (11.5)	66.7 (12.6)	-	5.1
**Man β(1-4) GlcNAc**	ϕ	ψ	ω	**Pop (%)**
Cluster 1	-71.3 (11.7)	-122.2 (14.1)	-	94.8
Cluster 2	-72.4 (10.9)	70.3 (11.3)	-	3.2
Cluster 3	-167.7 (11.9)	-145.0 (8.1)	-	2.2
**Man α(1-3) Man (1-3)**	ϕ	ψ	ω	**Pop (%)**
Cluster 1	72.2 (9.0)	141.3 (14.6)	-	58.5
Cluster 2	71.2 (10.4)	96.9 (10.9)	-	41.5
**Man α(1-3) Man (1-6)**	ϕ	ψ	ω	**Pop (%)**
Cluster 1	75.8 (11.6)	143.9 (16.7)	-	53.5
Cluster 2	70.2 (10.7)	99.9 (9.5)	-	26.5
Cluster 2	70.2 (10.7)	150.2 (10.0)	-	19.9
**Man α(1-6) Man**	ϕ	ψ	ω	**Pop (%)**
Cluster 1	69.5 (12.3)	-173.0 (15.5)	54.1 (12.6)	55.3
Cluster 2	66.3 (9.8)	-178.0 (12.6)	-176.1 (12.1)	38.2
Cluster 3	81.4 (13.1)	88.3 (11.3)	49.3 (10.3)	4.8
Cluster 4	82.0 (8.3)	-76.0 (10.9)	-148.8 (10.6)	0.0
Cluster 5	75.0 (7.31)	-171.1 (9.5)	-64.8 (8.2)	1.7
**Man α(1-6) Man (1-6)**	ϕ	ψ	ω	**Pop (%)**
Cluster 1	70.3 (10.4)	-171.5 (15.8)	54.7 (10.7)	74.9
Cluster 2	71.2 (7.4)	-178.9 (14.1)	-73.2 (8.8)	5.4
Cluster 3	69.4 (7.2)	-117.9 (12.3)	-67.0 (9.7)	9.4
Cluster 4	69.8 (9.2)	-175.0 (19.6)	-164.4 (11.3)	6.2
Cluster 5	70.3 (10.6)	83.9 (12.5)	48.0 (9.3)	4.1
**Man α(1-2) Man (1-3)**	ϕ	ψ	ω	**Pop (%)**
Cluster 1	75.4 (9.0)	151.7 (15.6)	-	70.8
Cluster 2	71.1 (9.7)	105.9 (11.6)	-	29.2
**Man α(1-2) Man (1-3)**	ϕ	ψ	ω	**Pop (%)**
Cluster 1	73.9 (8.8)	151.2 (15.2)	-	74.8
Cluster 2	70.1 (9.2)	106.6 (11.7)	-	25.2
**Man α(1-2) Man (1-6)**	ϕ	ψ	ω	**Pop (%)**
Cluster 1	73.9 (8.8)	151.2 (15.2)	-	74.8
Cluster 2	70.1 (9.2)	106.6 (11.7)	-	25.2

11 Man 8 II

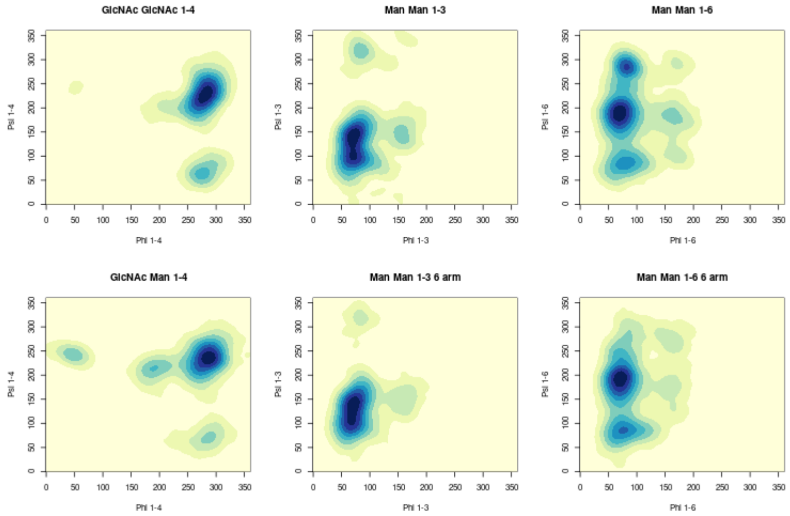

Figure S. 11: 2D Kernel density estimates for the ϕ / ψ angle distributions for Man 8 II.

Table S. 10: The ϕ / ψ / ω angle distributions for Man 8 II.

**GlcNAc β(1-4) GlcNAc**	ϕ	ψ	ω	**Pop (%)**
Cluster 1	-79.4 (11.4)	-132.7 (17.5)	-	95.4
Cluster 2	-82.4 (11.2)	65.6 (11.0)	-	4.6
**Man β(1-4) GlcNAc**	ϕ	ψ	ω	**Pop (%)**
Cluster 1	-75.1 (12.4)	-125.0 (14.9)	-	93.9
Cluster 2	-72.8 (8.2)	69.6 (8.0)	-	1.1
Cluster 3	-167.9 (12.5)	-145.3 (8.2)	-	3.3
Cluster 4	47.9 (8.5)	-117.23 (5.7)	-	1.7
**Man α(1-3) Man (1-3)**	ϕ	ψ	ω	**Pop (%)**
Cluster 1	72.2 (9.1)	141.7 (14.6)	-	61.0
Cluster 2	71.0 (10.4)	97.0 (10.8)	-	39.0
**Man α(1-3) Man (1-6)**	ϕ	ψ	ω	**Pop (%)**
Cluster 1	72.1 (9.2)	139.7 (14.8)	-	64.7
Cluster 2	67.4 (9.6)	100.2 (10.3)	-	35.3
**Man α(1-6) Man**	ϕ	ψ	ω	**Pop (%)**
Cluster 1	71.1 (10.8)	-171.6 (16.3)	55.4 (11.2)	47.5
Cluster 2	67.4 (9.9)	-173.9 (14.9)	-175.6 (12.0)	18.0
Cluster 3	79.6 (15.1)	85.7 (13.3)	48.9 (10.4)	11.8
Cluster 4	81.9 (7.6)	- 74.7 (9.6)	-150.7 (9.4)	16.6
Cluster 5	72.6 (8.9)	-164.8 (19.3)	- 67.6 (11.7)	6.0
**Man α(1-6) Man (1-6)**	ϕ	ψ	ω	**Pop (%)**
Cluster 1	71.4 (10.2)	-168.5 (16.4)	54.8 (10.3)	67.5
Cluster 2	69.4 (8.8)	-175.8 (13.9)	- 80.6 (13.5)	5.0
Cluster 3	69.1 (6.3)	-119.2 (8.8)	- 63.6 (7.6)	2.4
Cluster 4	69.5 (9.2)	-175.5 (21.3)	-166.7 (10.9)	6.7
Cluster 5	79.0 (16.6)	86.2 (11.7)	50.1 (9.8)	13.1
Cluster 6	71.6 (10.6)	81.9 (11.4)	-166.1 (13.5)	5.4
**Man α(1-2) Man (1-3)**	ϕ	ψ	ω	**Pop (%)**
Cluster 1	74.7 (9.0)	152.3 (15.5)	-	62.9
Cluster 2	72.2 (10.0)	97.8 (17.8)	-	37.1
**Man α(1-2) Man (1-3)**	ϕ	ψ	ω	**Pop (%)**
Cluster 1	74.3 (8.4)	152.1 (15.1)	-	75.6
Cluster 2	70.0 (9.2)	106.6 (11.7)	-	24.4
**Man α(1-2) Man (1-6)(1-6)**	ϕ	ψ	ω	**Pop (%)**
Cluster 1	74.3 (8.8)	152.1 (15.2)	-	75.6
Cluster 2	70.0 (9.2)	106.7 (11.7)	-	24.4

12 Man 8 III

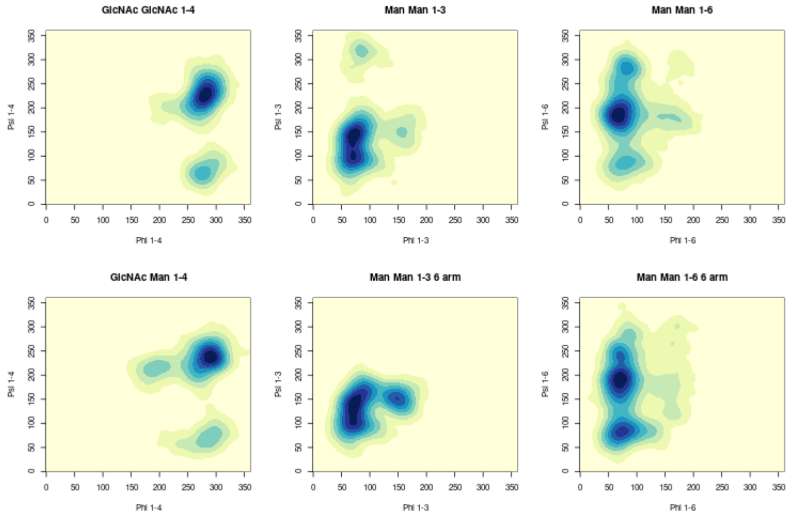

Figure S. 12: 2D Kernel density estimates for the ϕ / ψ angle distributions for Man 8 III.

Table S. 11: The ϕ / ψ / ω angle distributions for Man 8 III.

**GlcNAc β(1-4) GlcNAc**	ϕ	ψ	ω	**Pop (%)**
Cluster 1	-79.3 (10.5)	-132.7 (16.4)	-	94.0
Cluster 2	-82.3 (10.6)	65.9 (11.1)	-	6.0
**Man β(1-4) GlcNAc**	ϕ	ψ	ω	**Pop (%)**
Cluster 1	-71.8 (11.9)	-123.4 (13.9)	-	94.0
Cluster 2	-71.6 (10.3)	69.8 (10.5)	-	3.2
Cluster 3	-167.7 (11.2)	-145.4 (7.7)	-	2.7
**Man α(1-3) Man (1-3)**	ϕ	ψ	ω	**Pop (%)**
Cluster 1	72.4 (9.0)	142.1 (14.1)	-	61.2
Cluster 2	71.8 (9.8)	97.3 (10.2)	-	37.8
**Man α(1-3) Man (1-6)**	ϕ	ψ	ω	**Pop (%)**
Cluster 1	75.4 (11.1)	143.5 (16.2)	-	58.3
Cluster 2	71.1 (10.2)	100.6 (9.1)	-	27.7
Cluster 3	148.3 (9.3)	151.5 (8.7)	-	14.1
**Man α(1-6) Man**	ϕ	ψ	ω	**Pop (%)**
Cluster 1	70.6 (11.7)	-172.7 (14.8)	56.2 (11.7)	36.7
Cluster 2	66.3 (9.8)	-178.4 (12.7)	-176.7 (11.7)	35.9
Cluster 3	80.2 (11.7)	86.2 (9.7)	48.0 (9.3)	4.3
Cluster 4	82.9 (8.0)	- 76.2 (9.39)	-150.8 (9.31)	8.1
Cluster 5	74.5 (9.7)	-152.8 (16.7)	- 64.6 (11.8)	13.0
Cluster 6	72.4 (7.9)	-100.0 (8.2)	- 72.1 (8.0)	2.2
**Man α(1-6) Man (1-6)**	ϕ	ψ	ω	**Pop (%)**
Cluster 1	71.2 (10.1)	-170.4 (16.0)	55.1 (10.3)	50.0
Cluster 2	70.2 (8.5)	-178.3 (14.4)	- 76.2 (10.7)	5.9
Cluster 3	69.9 (6.8)	-117.7 (10.2)	- 66.0 (8.5)	8.2
Cluster 4	69.5 (8.4)	179.8 (16.4)	-166.4 (9.6)	5.9
Cluster 5	79.4 (15.8)	85.4 (11.0)	49.5 (9.4)	12.0
Cluster 6	71.7 (11.3)	81.4 (11.8)	-166.5 (14.2)	18.0
**Man α(1-2) Man (1-3)**	ϕ	ψ	ω	**Pop (%)**
Cluster 1	73.7 (8.6)	149.5 (14.3)	-	70.4
Cluster 2	69.7(8.9)	107.5 (11.3)	-	29.5
**Man α(1-2) Man (1-6)(1-3)**	ϕ	ψ	ω	**Pop (%)**
Cluster 1	72.3 (7.9)	149.5 (13.3)	-	72.2
Cluster 2	69.9 (8.1)	111.1 (10.3)	-	27.8
**Man α(1-2) Man (1-6)(1-6)**	ϕ	ψ	ω	**Pop (%)**
Cluster 1	72.3 (7.9)	149.5 (13.3)	-	72.2
Cluster 2	69.1 (8.1)	111.0 (10.4)	-	27.8

13 Man 9

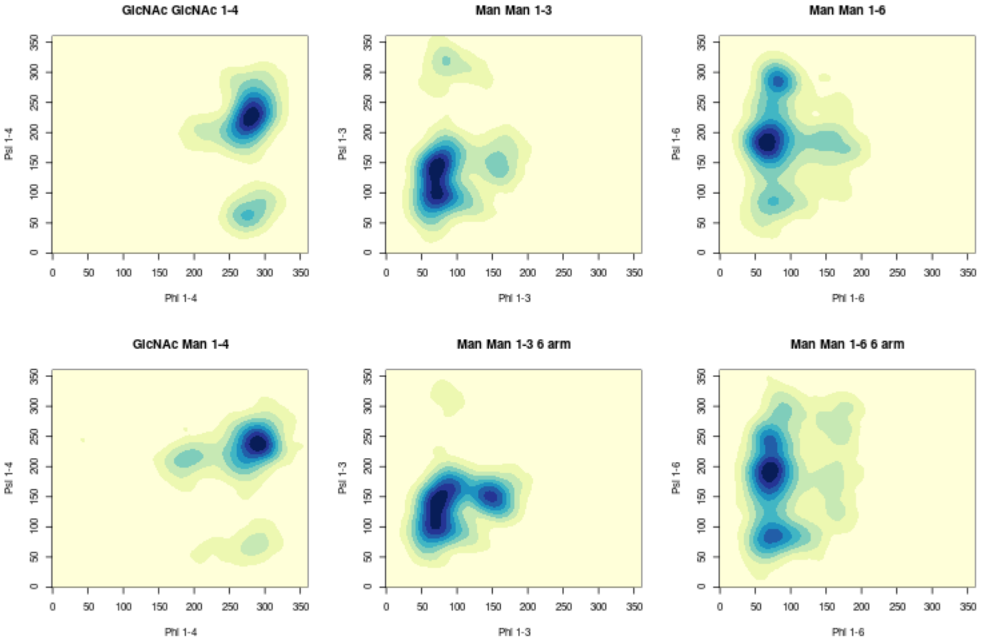

Figure S. 13: 2D Kernel density estimates for the ϕ / ψ angle distributions for Man 8.

Table S. 12: The ϕ / ψ / ω angle distributions for Man 9

**GlcNAc β(1-4) GlcNAc**	ϕ	ψ	ω	**Pop (%)**
Cluster 1	-78.8 (10.5)	-131.7 (17.8)	-	95.8
Cluster 2	-82.3 (11.8)	65.9 (11.5)	-	4.2
**Man β(1-4) GlcNAc**	ϕ	ψ	ω	**Pop (%)**
Cluster 1	-71.8 (11.7)	-123.1 (13.5)	-	97.5
Cluster 3	-168.4 (12.5)	-146.1 (8.7)	-	2.5
**Man α(1-3) Man (1-3)**	ϕ	ψ	ω	**Pop (%)**
Cluster 1	72.3 (9.2)	141.5 (14.7)	-	58.5
Cluster 2	71.4 (10.8)	96.7 (11.1)	-	41.5
**Man α(1-3) Man (1-6)**	ϕ	ψ	ω	**Pop (%)**
Cluster 1	76.1 (12.1)	144.8 (16.9)	-	55.4
Cluster 2	69.8 (10.9)	100.2 (9.7)	-	26.4
Cluster 2	147.8 (10.9)	150.8 (10.4)	-	18.2
**Man α(1-6) Man**	ϕ	ψ	ω	**Pop (%)**
Cluster 1	70.3 (12.6)	-173.3 (15.5)	55.1 (12.4)	38.0
Cluster 2	66.0 (9.8)	-178.6 (13.0)	-176.9 (11.6)	37.2
Cluster 3	77.9 (11.2)	86.9 (10.5)	46.2 (9.3)	2.8
Cluster 4	82.0 (7.5)	-74.4 (9.6)	-151.1 (9.6)	11.9
Cluster 5	73.2 (9.4)	-157.3 (20.6)	-65.4 (11.3)	7.0
Cluster 6	75.9 (8.6)	-95.6 (9.7)	-74.8 (8.9)	1.8
Cluster 7	147.3 (9.3)	-171.6 (7.8)	-151.1 (7.4)	1.3
**Man α(1-6) Man (1-6)**	ϕ	ψ	ω	**Pop (%)**
Cluster 1	71.4 (10.2)	-168.8 (16.6)	57.6 (10.3)	56.9
Cluster 2	71.2 (6.7)	-180.0 (14.6)	-73.2 (7.8)	3.8
Cluster 3	70.2 (7.4)	-118.1 (11.7)	-67.4 (10.0)	11.6
Cluster 4	69.2 (9.5)	-179.3 (22.9)	-165.7 (11.4)	7.5
Cluster 5	78.8 (15.92)	86.3 (11.8)	50.8 (9.65)	4.1
Cluster 6	72.6 (11.8)	82.9 (11.8)	-163.9 (17.6)	10.0
**Man α(1-2) Man (1-3)**	ϕ	ψ	ω	**Pop (%)**
Cluster 1	75.4 (9.2)	151.8 (15.7)	-	69.6
Cluster 2	71.1 (9.1)	105.9 (11.5)	-	30.4
**Man α(1-2) Man (1-3)**	ϕ	ψ	ω	**Pop (%)**
Cluster 1	74.1 (8.8)	151.7 (15.4)	-	74.7
Cluster 2	70.1 (9.6)	106.3 (11.8)	-	25.3
**Man α(1-2) Man (1-6)**	ϕ	ψ	ω	**Pop (%)**
Cluster 1	74.1 (8.8)	151.8 (15.4)	-	74.7
Cluster 2	70.1 (9.6)	106.3 (11.7)	-	25.3
**Man α(1-2) Man (1-6)(1-6)**	ϕ	ψ	ω	**Pop (%)**
Cluster 1	74.4 (8.8)	151.2 (15.1)	-	76.5
Cluster 2	70.5 (9.5)	106.6 (12.2)	-	23.5

14 FcγRC: Man5 N45

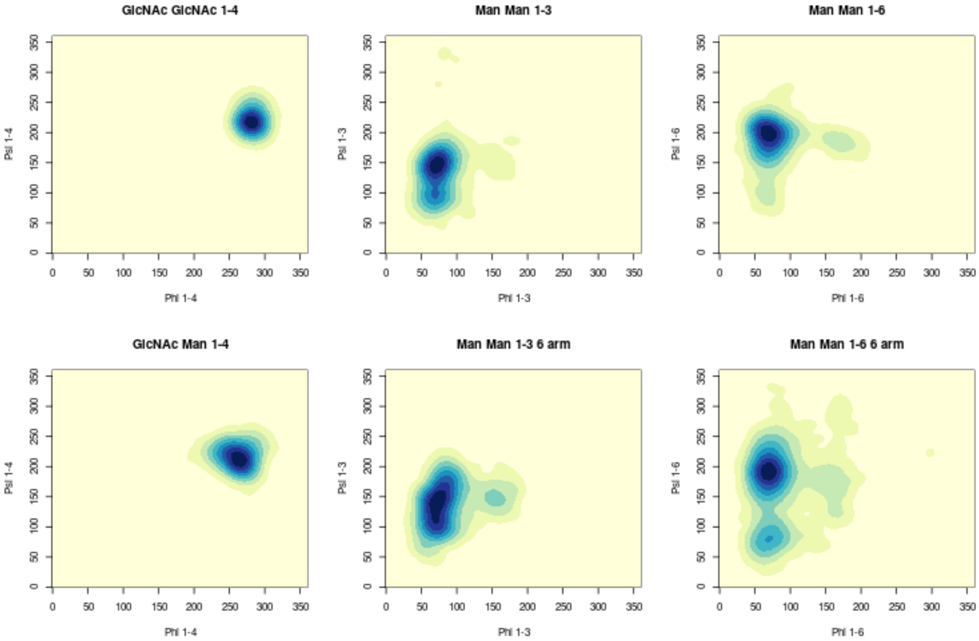

Figure S. 14: 2D Kernel density estimates for the ϕ / ψ angle distributions for Man 5 (N45).

Table S. 13: The ϕ / ψ / ω angle distributions for Man 5 on Fcγ-RC N45

**GlcNAc β(1-4) GlcNAc**	ϕ	ψ	ω	**Pop (%)**
Cluster 1	-78.62 (6.6)	-142.4 (9.5)	-	100
**Man β(1-4) GlcNAc**	ϕ	ψ	ω	**Pop (%)**
Cluster 1	-96.9 (10.7)	-146.3 (11.3)	-	93.0
**Man α(1-3) Man (1-3)**	ϕ	ψ	ω	**Pop (%)**
Cluster 1	72.1 (8.1)	145.9 (12.7)	-	82.9
Cluster 2	68.2 (8.4)	97.2 (10.1)	-	17.1
**Man α(1-3) Man (1-6)**	ϕ	ψ	ω	**Pop (%)**
Cluster 1	75.4 (9.5)	147.4 (15.0)	-	65.9
Cluster 2	70.6 (8.3)	108.72 (10.6)	-	34.1
**Man α(1-6) Man**	ϕ	ψ	ω	**Pop (%)**
Cluster 1	69.1 (9.5)	-162.1 (13.9)	-172.2 (10.6)	100
**Man α(1-6) Man (1-6)**	ϕ	ψ	ω	**Pop (%)**
Cluster 1	69.7 (10.2)	-172.2 (15.1)	53.8 (10.7)	44.2
Cluster 2	69.2 (9.4)	-166.3 (15.3)	-87.0 (15.8)	36.9
Cluster 3	70.6 (10.3)	-155.6 (18.9)	-161.1 (10.6)	12.3
Cluster 4	69.2 (10.6)	79.9 (11.5)	-166.9 (12.5)	6.7

15 FcγRC: Man5 N162

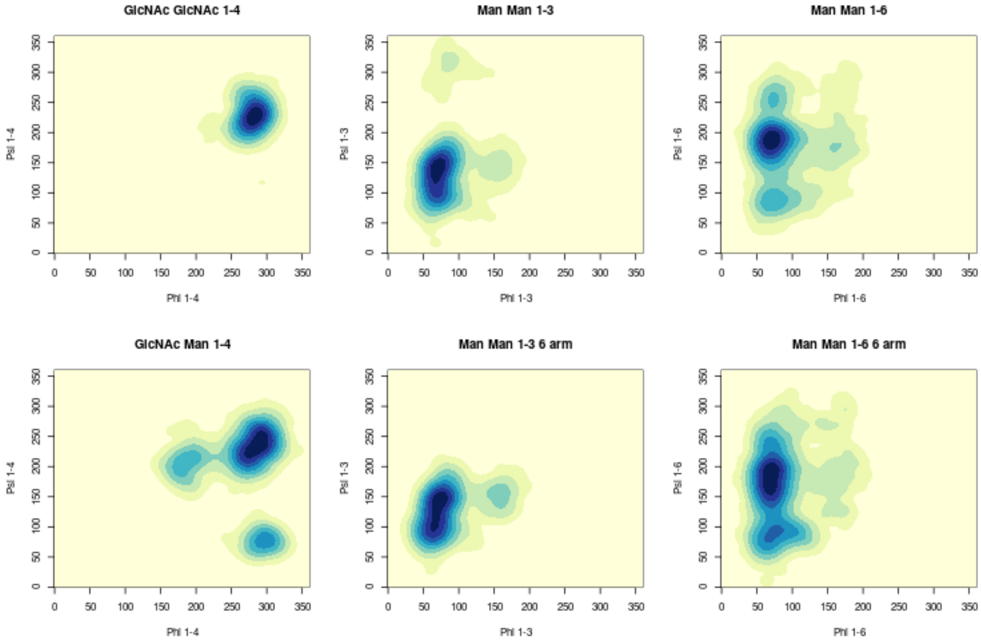

Figure S. 15: 2D Kernel density estimates for the ϕ / ψ angle distributions for Man 5 on Fcγ-RC N162.

Table S. 14: The ϕ / ψ / ω angle distributions for Man 5 on Fcγ-RC N162

**Man α(1-6) Man (1-6)**	ϕ	ψ	ω	**Pop (%)**
Cluster 1	-78.2 (9.3)	-132.8 (14.9)	-	100
**Man β(1-4) GlcNAc**	ϕ	ψ	ω	**Pop (%)**
Cluster 1	-74.12 (12.9)	-125.7 (17.2)	-	86.7
Cluster 2	-62.9 (9.6)	76.7 (8.9)	-	8.3
Cluster 3	-175.3 (10.8)	-152.2 (11.8)	-	5.0
**Man α(1-3) Man (1-3)**	ϕ	ψ	ω	**Pop (%)**
Cluster 1	70.9 (8.8)	141.5 (13.9)	-	72.0
Cluster 2	68.7 (8.9)	100.9 (10.0)	-	28.0
**Man α(1-3) Man (1-6)**	ϕ	ψ	ω	**Pop (%)**
Cluster 1	73.5 (8.9)	144.5 (11.1)	-	52.1
Cluster 2	65.9 (9.4)	101.9 (13.6)	-	47.9
**Man α(1-6) Man**	ϕ	ψ	ω	**Pop (%)**
Cluster 1	69.1 (11.2)	-175.0 (16.1)	52.3 (11.9)	9.6
Cluster 2	71.6 (11.9)	-172.8 (16.7)	-169.3 (14.9)	82.5
Cluster 3	74.0 (13.3)	87.6,(12.8)	52.6 (11.7)	7.8
**Man α(1-6) Man (1-6)**	ϕ	ψ	ω	**Pop (%)**
Cluster 1	70.3 (10.1)	179.1 (22.7)	57.4 (11.0)	81.1
Cluster 2	75.1 (15.8)	89.4 (12.6)	53.1 (9.7)	18.9

16 FcγRC: Man9 N45

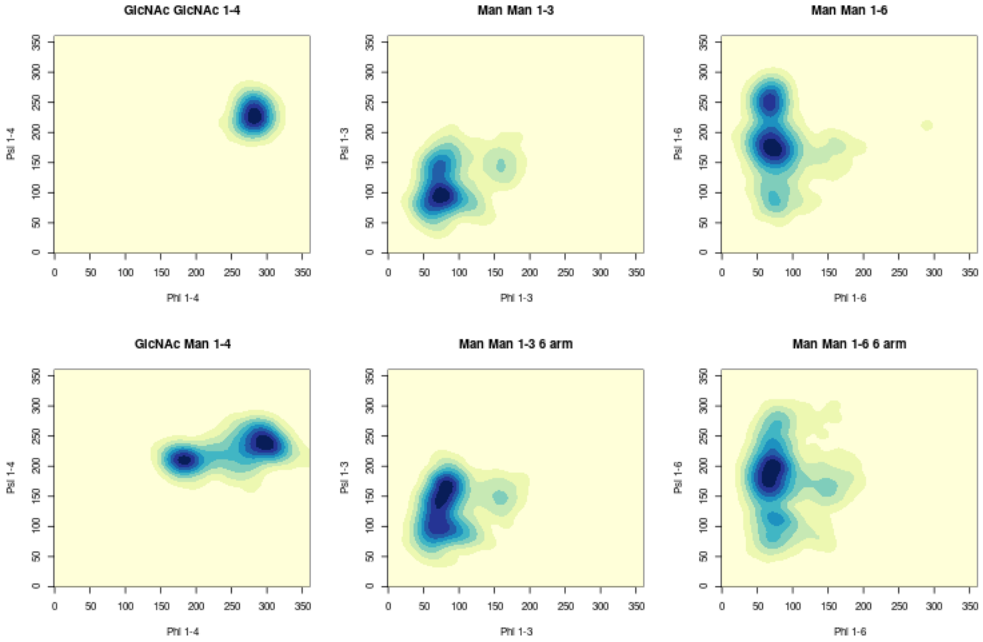

Figure S. 16: 2D Kernel density estimates for the ϕ / ψ angle distributions for N45.

Table S. 15: The ϕ / ψ / ω angle distributions for Man 9 on Fcγ-RC N45

**GlcNAc β(1-4) GlcNAc**	ϕ	ψ	ω	**Pop (%)**
Cluster 1	-77.9 (7.5)	-132.6 (11.8)	-	100
**Man β(1-4) GlcNAc**	ϕ	ψ	ω	**Pop (%)**
Cluster 1	-65.9 (14.1)	-122.3 (11.6)	-	64.6
Cluster 2	-176.2 (12.7)	-149.6 (7.1)	-	35.4
**Man α(1-3) Man (1-3)**	ϕ	ψ	ω	**Pop (%)**
Cluster 1	72.2 (7.9)	138.1 (12.7)	-	23.1
Cluster 2	73.7 (11.4)	93.4 (11.1)	-	76.9
**Man α(1-3) Man (1-6)**	ϕ	ψ	ω	**Pop (%)**
Cluster 1	78.3 (9.1)	155.1 (15.3)	-	60.2
Cluster 2	70.8 (12.0)	101.0 (12.4)	-	39.8
**Man α(1-6) Man**	ϕ	ψ	ω	**Pop (%)**
Cluster 1	71.2 (11.5)	173.8 (13.1)	59.4 (15.2)	57.1
Cluster 2	71.8 (8.7)	-173.6 (14.9)	-75.0 (11.3)	15.1
Cluster 3	67.1 (7.2)	-111.6,(12.1)	-69.1 (12.4)	27.9
**Man α(1-6) Man (1-6)**	ϕ	ψ	ω	**Pop (%)**
Cluster 1	71.0 (9.8)	174.8 (14.8)	- 76.7 (14.1)	17.2
Cluster 2	65.7 (9.5)	-178.4 (22.2)	-170.2 (10.8)	33.9
Cluster 3	70.5 (10.2)	-164.1 (14.2)	56.4 (11.7)	42.9
Cluster 4	76.1 (9.4)	113.3 (8.3)	- 87.5 (7.9)	5.9
**Man α(1-2) Man (1-3)**	ϕ	ψ	ω	**Pop (%)**
Cluster 1	74.3 (8.4)	148.2 (13.2)	-	61.1
Cluster 2	72.7 (10.1)	105.3 (11.1)	-	38.9
**Man α(1-2) Man (1-3)**	ϕ	ψ	ω	**Pop (%)**
Cluster 1	73.2 (8.3)	149.3 (14.3)	-	69.1
Cluster 2	70.1 (8.5)	106.0 (10.9)	-	30.9
**Man α(1-2) Man (1-6)**	ϕ	ψ	ω	**Pop (%)**
Cluster 1	72.2 (7.6)	146.9 (12.4)	-	77.4
Cluster 2	69.9 (7.9)	107.1 (10.9)	-	22.6
**Man α(1-2) Man (1-6)(1-6)**	ϕ	ψ	ω	**Pop (%)**
Cluster 1	74.5 (9.0)	151.9 (13.6)	-	78.0
Cluster 2	72.2 (8.1)	104.2 (11.3)	-	22.0

17 FcγRC: Man9 N162

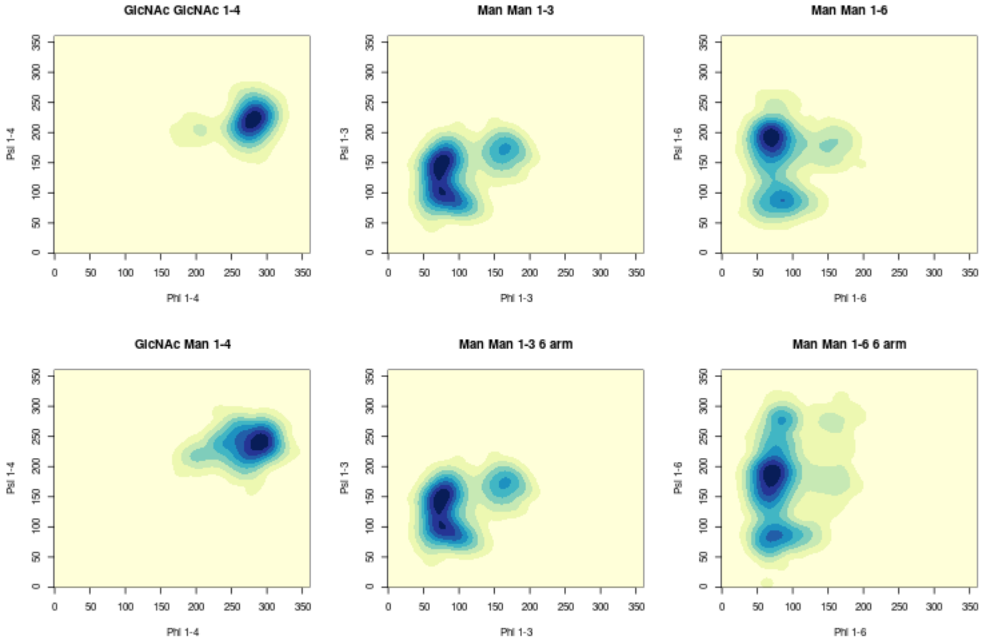

Figure S. 17: 2D Kernel density estimates for the ϕ / ψ angle distributions for Man9 on the Fcγ-RC N162

Table S. 16: The ϕ / ψ / ω angle distributions for Man 9 on Fcγ-RC N162

**GlcNAc β(1-4) GlcNAc**	ϕ	ψ	ω	**Pop (%)**
Cluster 1	-79.8 (9.6)	-138.9 (13.2)	-	100
**Man β(1-4) GlcNAc**	ϕ	ψ	ω	**Pop (%)**
Cluster 1	-75.1 (17.0)	-120.5 (13.8)	-	100
**Man α(1-3) Man (1-3)**	ϕ	ψ	ω	**Pop (%)**
Cluster 1	72.0 (9.2)	145.4(15.2)	-	58.7
Cluster 2	79.0 (12.6)	96.5 (10.4)	-	34.6
Cluster 3	163.7 (11.2)	170.9(12.0)	-	6.7
**Man α(1-3) Man (1-6)**	ϕ	ψ	ω	**Pop (%)**
Cluster 1	77.9 (9.4)	156.2 (17.2)	-	70.8
Cluster 2	70.3 (9.4)	108.8 (10.7)	-	29.2
**Man α(1-6) Man**	ϕ	ψ	ω	**Pop (%)**
Cluster 1	70.1 (10.6)	-170.6 (16.0)	54.6 (11.1)	82.6
Cluster 2	83.4 (14.3)	88.2 (12.2)	47.8 (9.5)	17.4
**Man α(1-6) Man (1-6)**	ϕ	ψ	ω	**Pop (%)**
Cluster 1	70.2 (10.9)	-172.8 (18.8)	54.8 (11.2)	52.8
Cluster 2	69.9 (9.7)	176.3 (19.8)	-76.6 (12.8)	9.5
Cluster 3	83.1 (8.0)	-85.2 (10.9)	-80.0 (10.1)	4.7
Cluster 4	74.6 (15.6	84.7 (11.1)	48.8 (10.8)	16.8
Cluster 5	69.9 (10.1)	175.5 (21.1)	-168.1 (14.0)	16.2
**Man α(1-2) Man (1-3)**	ϕ	ψ	ω	**Pop (%)**
Cluster 1	76.6 (8.3)	156.5 (13.8)	-	77.8
Cluster 2	68.5 (8.1)	100.1 (9.6)	-	22.2
**Man α(1-2) Man (1-3)**	ϕ	ψ	ω	**Pop (%)**
Cluster 1	74.4 (9.0)	154.8 (16.1)	-	93.9
Cluster 2	66.6 (4.8)	107.4 (5.2)	-	6.1
**Man α(1-2) Man (1-6)**	ϕ	ψ	ω	**Pop (%)**
Cluster 1	71.8 (7.6)	146.4 (12.9)	-	79.8
Cluster 2	70.0 (7.4)	111.1 (10.1)	-	20.2
**Man α(1-2) Man (1-6)(1-6)**	ϕ	ω	ψ	**Pop (%)**
Cluster 1	74.7 (8.7)	150.9 (14.9)	-	73.8
Cluster 2	70.4 (8.3)	105.3 (11.6)	-	26.2

18 DBSCAN Parameters

Dihedral Angle's	eps (ϵ)	Minimum points
GlcNAc β(1-4) GlcNAc	7.5	75
Man β(1-4) GlcNAc	7.5	100
Man α(1-3) Man (1-3)	10	1000
Man α(1-3) Man (1-6)	10	1000
Man α(1-6) Man	16	400
Man α(1-6) Man (1-6)	16	400
Man α(1-2) Man	10	1000
Man α(1-2) Man (2)	10	1000
Man α(1-2) Man (3)	10	1000
Man α(1-2) Man (4)	10	1000

Table S. 17: List of parameters used for the clustering analysis of Man 9. Note: some of these parameters may need to be tweaked as clusters can bleed into each other. This algorithm will take all RAM it's given, best practice to test on smaller data sets before scaling up to production datasets in order to prevent crashes.

19 Man 9 / 8(II) Distance Measurements

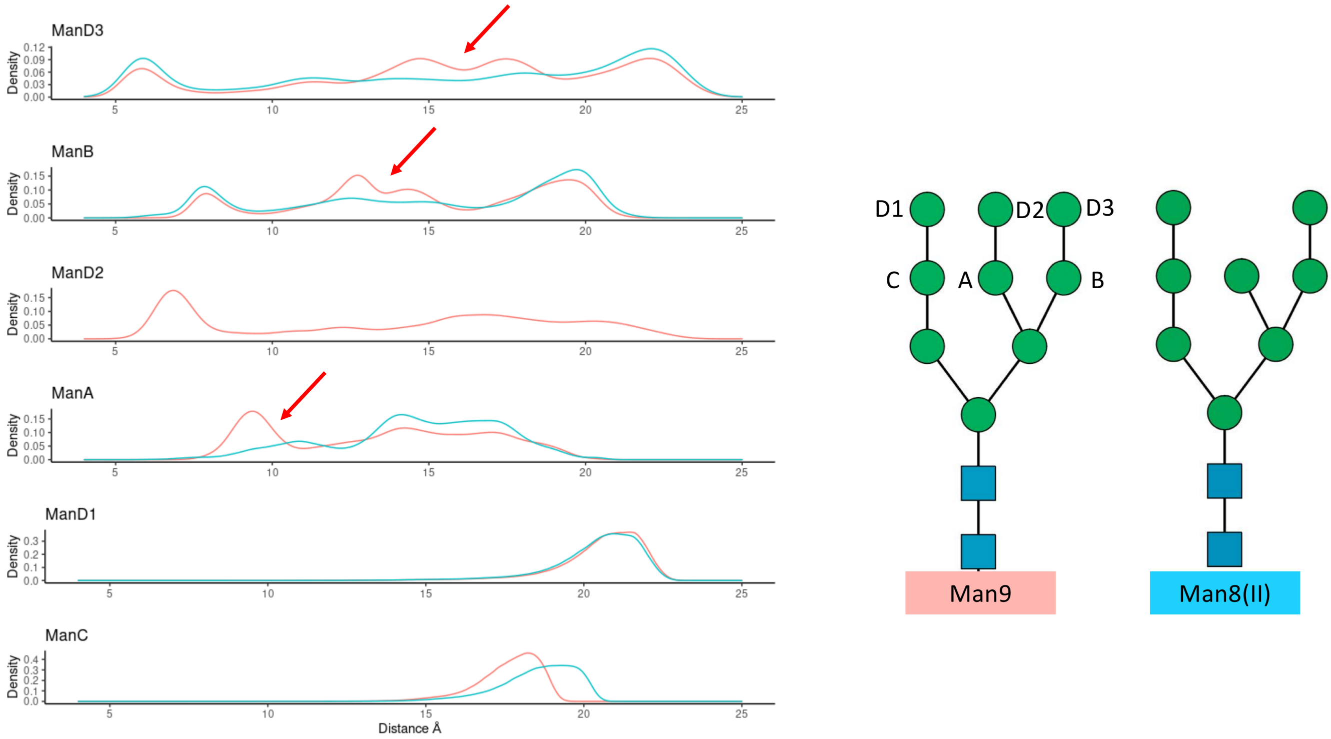

Figure S. 18: KDE analysis of the distances between the anomeric protons of the reducing GlcNAc and that of the specified mannose residues in the legend for Man9 (red) and Man8(II) (blue) obtained from our simulations. The red arrows highlight shorter distances only observed in Man9, which indicate the higher occurrence of folded structures, in agreement with a progressively higher occurrence of arm-arm interactions with arm elongation, as described in the main text. KDE analysis made with r and diagrams with RStudio (www.rstudio.com).
